# Association of Prior Atherosclerotic Cardiovascular Disease with Dementia After Stroke: A Retrospective Cohort Study

**DOI:** 10.3233/JAD-200536

**Published:** 2020-09-29

**Authors:** Zhirong Yang, Duncan Edwards, Stephen Burgess, Carol Brayne, Jonathan Mant

**Affiliations:** a Primary Care Unit, Department of Public Health and Primary Care, School of Clinical Medicine, University of Cambridge, Cambridge, UK; b MRC Biostatistics Unit, School of Clinical Medicine, University of Cambridge, Cambridge, UK; c Cardiovascular Epidemiology Unit, Department of Public Health and Primary Care, School of Clinical Medicine, University of Cambridge, Cambridge, UK; d Institute of Public Health, School of Clinical Medicine, University of Cambridge, Cambridge, UK

**Keywords:** Atherosclerotic cardiovascular disease, cohort study, coronary artery disease, dementia, peripheral arterial disease, stroke

## Abstract

**Background:**

Prior atherosclerotic cardiovascular disease (ASCVD), including coronary heart disease (CHD) and peripheral artery disease (PAD), are common among patients with stroke, a known risk factor for dementia. However, whether these conditions further increase the risk of post-stroke dementia remains uncertain.

**Objective:**

To examine whether prior ASCVD is associated with increased risk of dementia among stroke patients.

**Methods:**

A retrospective cohort study was conducted using the Clinical Practice Research Datalink with linkage to hospital data. Patients with first-ever stroke between 2006 and 2017 were followed up to 10 years. We used multi-variable Cox regression models to examine the associations of prior ASCVD with dementia and the impact of prior ASCVD onset and duration.

**Results:**

Among 63,959 patients, 7,265 cases (11.4%) developed post-stroke dementia during a median of 3.6-year follow-up. The hazard ratio (HR) of dementia adjusted for demographics and lifestyle was 1.18 (95% CI: 1.12–1.25) for ASCVD, 1.16 (1.10–1.23) for CHD, and 1.25 (1.13–1.37) for PAD. The HRs additionally adjusted for multimorbidity and medications were 1.07 (1.00–1.13), 1.04 (0.98–1.11), and 1.11 (1.00–1.22), respectively. Based on the fully adjusted estimates, there was no linear relationship between the age of ASCVD onset and post-stroke dementia (all p-trend >0.05). The adjusted risk of dementia was not increased with the duration of pre-stroke ASCVD (all p-trend >0.05).

**Conclusion:**

Stroke patients with prior ASCVD are more likely to develop subsequent dementia. After full adjustment for confounding, however, the risk of post-stroke dementia is attenuated, with only a slight increase with prior ASCVD.

## INTRODUCTION

Stroke patients have a higher risk of dementia than the general population [1, 2], with 10% to 15% developing dementia five years after stroke [3]. Whether there are differences between stroke survivors that influence their likelihood of developing dementia and whether preventive interventions might be effective is to date unclear [4], although dementia is associated with multiple cardiovascular risk factors in the general population [5].

Coronary heart disease (CHD) and peripheral artery disease (PAD), the two major manifestations of atherosclerotic cardiovascular disease (ASCVD), often co-exist in people with stroke [6] and are associated with risk of dementia in the general population [7–9]. Dementia may be a consequence of underlying atherosclerosis or could be independent of atherosclerosis as a convergent disease process sharing major pathophysiological elements, such as cholesterol, inflammation, and Apolipoprotein E polymorphism [10]. The myocardial dysfunction may contribute to subsequent cognitive impairment by affecting the maintenance of cerebral blood flow and cerebral perfusion homeostasis [11].

Previous research has shown a higher risk of future cardiovascular events in patients with stroke and prior ASCVD than those with stroke alone [12]. However, it is not clear whether the risk of dementia would also be increased in patients with stroke and prior ASCVD. A recent systematic review found that CHD and PAD were associated with dementia after stroke [13]. However, only two of the included studies provided adjusted estimates of the association, and there were other limitations of the included studies, including potential selection bias, small sample size, and lack of long-term follow-up (median of maximum follow-up: 0.5 year; interquartile range (IQR): 0.25 to 4 years) [13]. The two studies [14, 15] that adjusted for confounding only took into account demographic covariates and stroke severity but not other potential confounders of dementia, such as lifestyle, cardiovascular risk factors, co-morbidities, and medications[3, 4, 16]. It is not clear whether the association might vary with age at first diagnosis of prior ASCVD, with duration of prior ASCVD, or in different patient subgroups [13]. Whether the association of atherosclerosis with dementia varies by vessel affected (e.g., coronary arteries or peripheral arteries) is not known for the general population [17], let alone in stroke patients [13]. Understanding the roles of prior ASCVD in post-stroke dementia may help inform the monitoring and prevention strategies for dementia in these patients.

We conducted this study to examine the overall association of prior ASCVD with dementia after stroke and explore whether the association differs by age at first diagnosis of prior ASCVD, by duration of prior ASCVD, or in different patient subgroups.

## MATERIALS AND METHODS

### Data source

We conducted a retrospective cohort study using the Clinical Practice Research Datalink (CPRD), which provides anonymized data extracted from primary care medical records (including nursing home patients), with coverage of a representative sample of approximately 7% of the UK population from more than 670 general practices [18]. Patient-level data from the general practices (about 58% of all UK CPRD practices) which had consented to participate in the CPRD linkage scheme were linked to other existing data sources via a trusted third party (the Health and Social Care Information Centre). Where possible, we linked the CPRD data by unique patient identifier to Hospital Episode Statistics (HES), Death registration data from the Office for National Statistics (ONS), and Index of Multiple Deprivation (IMD).

### Study population

We included patients aged at least 18 years with a diagnosis of first-ever stroke (ischemic stroke or intracerebral hemorrhage) recorded in the CPRD between 1 January 2006 and 31 December 2017 (Codes for stroke was listed in Supplementary Table 1). To ensure quality of recording of pre-existing diagnoses and medications, eligible patients were also required to have at least 12-month record information before the index date of stroke. We excluded patients with any dementia codes prior to the index stroke.

### Post-stroke dementia

We defined post-stroke dementia as any type of dementia first recorded after the index stroke. In a sensitivity analysis, we further excluded patients having a first record of dementia within 6 months of the stroke to reduce the possibility of reverse causality. Dementia was identified using Read codes recorded in the CPRD and ICD-10 codes recorded in HES and ONS (Codes for dementia are listed in Supplementary Table 2).

### Exposure

The exposure of interest in this study is atherosclerosis manifested by CHD and PAD. We defined the two conditions as any related Read codes or ICD-10 codes recorded before the index stroke (Codes for CHD and PAD are listed in Supplementary Tables 3 and 4, respectively). We defined ASCVD as the presence of CHD or PAD. Patients with ASCVD were then classified into mutually exclusive categories according to the ASCVD type (CHD plus PAD, CHD only, and PAD only). We further considered as exposure level the age of first diagnosis of ASCVD (stratified into three age groups: young adults, <45 years; mid-life, 45 to 65; late life, >65) and length of pre-stroke ASCVD history (tertiles).

### Potential confounders

We included potential confounders representing demographics, lifestyle, cardiovascular factors, neuropsychological conditions, markers of immunity/inflammation, health care utilization, and medications. Demographic variables included age, gender, and socioeconomic status. Age was calculated on the date of index stroke and categorized into four groups (18–64, 65–74, 75–84 and ≥85 years) for subgroup analysis. Index of Multiple Deprivation (IMD) grouped by quintile was used as an indicator of socioeconomic status. The IMD includes seven domains: income; employment; health and disability; education, skills and training; barriers to housing and services; crime; and living environment. Where patient-level IMD was missing, we used the general practice-level IMD. Smoking status was classified as current, former, or never smoker. Body mass index (BMI) was analyzed as a continuous variable. For both smoking and BMI variables, the most recent data before the index stroke were used. Stroke subtype was classified into ischemic and hemorrhagic stroke (specific codes relating to ischemic stroke or unspecified stroke codes were regarded as ischemic stroke, considering these patients shared similar characteristics [19] and 90% of stroke in the UK is ischemic stroke [20]). Prior comorbidity was defined as the presence of any relevant Read code or ICD-10 code before the index stroke. These conditions included atrial fibrillation, alcohol problem, anxiety, asthma, chronic obstructive pulmonary disease, depression, diabetes, epilepsy, heart failure, hearing loss, hyperlipidemia, hypertension, Parkinson’s disease, rheumatoid arthritis, and transient ischemic attack. The codes for each condition were developed as part of a project developing the Cambridge Multimorbidity Score[21] and are publicly available on the website: http://www.phpc.cam.ac.uk/pcu/cprd_cam/codelists/. We used number of consultations within 365 days before index stroke as a measure of healthcare utilization. Pre-stroke medications, including statins, other lipid-lowering drugs, anticoagulant, antiplatelet, antihypertensive drugs, and antidiabetic drugs, were defined as any BNF codes recorded during the 365 days prior to index stroke. For comorbidity and medication, an absence of related codes was regarded as an absence of the condition.

### Statistical analysis

We described baseline characteristics and calculated incidence rate of dementia for the overall stroke cohort and then separately by prior ASCVD history.

We estimated the hazard ratios (HR) with 95% confidence intervals (CI) for dementia over 10 years using cause-specific Cox proportional hazards models, in which death as a competing-risks event was treated as being censored. Robust standard errors that allow for intragroup correlation were used to account for possible clustering effects by general practice. Follow-up started from the index stroke until any dementia occurrence or censoring, whichever occurred earlier. Censoring included death, transfer-out from the CPRD, the end of 10-year follow-up, or the last update of CPRD (31 July 2018). The proportional hazard assumption was examined by a log-log plot and by testing the significance level of the interaction terms between ASCVD and time over 10 years in our primary model. The primary model was conducted with full adjustment for demographics, lifestyle, comorbidity, healthcare utilization, and pre-stroke medications. To explore how the association of interest was influenced by the potential confounders, we also conducted two nested models with adjustment for parts of these confounder sets (demographics, lifestyle, and comorbidity; and demographics and lifestyle alone). Numerical variables (age, BMI, and consultation) were treated as cubic spline variables with four knots in all the models to accommodate their potential non-linear relationships with dementia [22]. All these models in our main analysis were complete-case analysis.

We then estimated the HR of dementia for each age group of first ASCVD diagnosis and each tertile of pre-stroke ASCVD duration. Only including patients with ASCVD, we tested the linear trend of the age of ASCVD onset and the length of pre-stroke ASCVD history associated with dementia by assigning the median values to each category and treating them as a numerical variable in the models [23]. We examined multiplicative and additive interactions between CHD and PAD by including their product term in the models and testing the product term and relative excess risk due to interaction, respectively [24].

To examine the robustness of the study results, we conducted a series of sensitivity analyses: 1) excluding patients having a first record of dementia within the first 6 months after stroke; 2) separating any unspecified stroke from ischemic stroke; 3) restricting to patients with linkage to HES data; 4) changing the missing value of BMI to the 5th or 95th percentile value and the missing value of smoking to never or current smoking, respectively; 5) using competing-risks regression models treating death as a competing-risks event.

Subgroup analysis by age group of stroke diagnosis, gender, stroke subtype, cardiovascular comorbidity, and pre-stroke statin use was then conducted using the primary full model. We examined the significance of interaction terms between the stratifying variable and ASCVD.

All the data management and statistical analyses were conducted using Stata 15. Quality control was performed before analysis (Supplementary Table 5). The statistical significance was *p* < 0.05 two-tailed except for subgroup analysis, where a Bonferroni correction was applied to the significance level that divided 0.05 by 11 subgroups examined (i.e., 0.0045).

We repeated the same analyses as above for CHD and PAD separately. We reported the results according to the RECORD statement (in the Supplementary Checklist) [25].

### Ethics approval and patient consents

Ethics approval was obtained from the Independent Scientific Advisory Committee of the CPRD (protocol number 17_201R), with no written consent from participants required.

## RESULTS

A total of 63,959 patients were included, of whom 16,900 (26.4%), 14,880 (23.3%), and 3,886 (6.1%) had prior ASCVD, CHD, and PAD, respectively (Supplementary Figure 1). The median age was 75 years (IQR: 64–83) and nearly half (49.2%) were women (Table 1, Supplementary Table 6). The median age at first pre-stroke diagnosis of ASCVD, CHD and PAD was 66 years (IQR: 58–75), 66 years (IQR: 57–75), and 69 years (IQR: 61–77), respectively. The median duration of ASCVD, CHD, and PAD before stroke was 10 years (IQR: 5–17), 11 years (IQR: 5–17), and 7 years (IQR: 4–12), respectively. Patients with complete baseline information (*n* = 57,902) were more likely to suffer comorbidities and receive pre-stroke medications (Supplementary Table 7).

**Table 1:** Baseline characteristics

	Atherosclerotic cardiovascular disease, number (%)				Total, number (%)	
	Presence		Absence	
Number of patients	16,900		47,059		63,959
Age, median (IQR)	78	(70–84)	73	(62–82)	75	(64–83)
Female	7,357	(43.5)	24,100	(51.2)	31,457	(49.2)
IMD Group 1 (least deprived)	3,330	(19.7)	10,438	(21.3)	13,398	(20.9)
Group 2	3,168	(18.8)	9,122	(18.6)	11,951	(18.7)
Group 3	3,536	(20.9)	10,675	(21.7)	13,778	(21.5)
Group 4	3,470	(20.5)	9,974	(20.3)	13,023	(20.4)
Group 5	3,396	(20.1)	8,870	(18.1)	11,809	(18.5)
Smoking Current ^a^	2,960	(17.5)	9,665	(20.5)	12,625	(19.7)
Former	6,987	(41.3)	13,788	(29.3)	20,775	(32.5)
Never	6,905	(40.9)	23,257	(49.4)	30,162	(47.2)
BMI, median (IQR) ^b^	26.9	(23.8–30.4)	26.5	(23.5–30.1)	26.6	(23.6–30.1)
Ischemic stroke ^c^	15,653	(92.6)	42,724	(90.8)	58,377	(91.3)
Intracerebral hemorrhage	1,247	(7.4)	4,335	(9.2)	5,582	(8.7)
Atrial fibrillation	5,275	(31.2)	7,624	(16.2)	12,899	(20.2)
Alcohol problems	824	(4.9)	2,215	(4.7)	3,039	(4.8)
Anxiety	3,398	(20.1)	8,743	(18.6)	12,141	(19.0)
Asthma	2,671	(15.8)	5,582	(11.9)	8,253	(12.9)
COPD	2,578	(15.3)	3,632	(7.7)	6,210	(9.7)
Coronary heart disease	14,880	(88.0)	0	(0)	14,880	(23.3)
Depression	4,949	(29.3)	12,025	(25.6)	16,974	(26.5)
Diabetes	4,776	(28.3)	6,653	(14.1)	11,429	(17.9)
Epilepsy	521	(3.1)	1,499	(3.2)	2020	(3.2)
Hearing loss	4,526	(26.8)	9,468	(20.1)	13,994	(21.9)
Heart failure	3,516	(20.8)	1,956	(4.2)	5,472	(8.6)
Hyperlipidaemia	7,742	(45.8)	9,901	(21.0)	17,643	(27.6)
Hypertension	12,321	(72.9)	24,988	(53.1)	37,309	(58.3)
Parkinson’s disease	274	(1.6)	554	(1.2)	828	(1.3)
Peripheral artery disease	3,886	(23.0)	0	(0)	3,886	(6.1)
Rheumatoid arthritis	1,403	(8.3)	2,782	(5.9)	4,185	(6.5)
Transient ischemic attack	2,098	(12.4)	4,864	(10.3)	6,962	(10.9)
Consultation, median (IQR)	44	(30–62)	29	(17–45)	33	(20–50)
Statins	11,871	(70.2)	13,530	(28.8)	25,401	(39.7)
Other lipid-lowering drugs	1,105	(6.5)	763	(1.6)	1,868	(2.9)
Anticoagulant	2,185	(12.9)	2,732	(5.8)	4,917	(7.7)
Antidiabetic drugs	3,650	(21.6)	4,896	(10.4)	8,546	(13.4)
Antihypertensive drugs	14,711	(87.0)	26,215	(55.7)	40,926	(64.0)
Antiplatelet	12,214	(72.3)	13,221	(28.1)	25,435	(39.8)

^a^A total of 397 (0.62%) patients had missing value of smoking status: 48 (0.28%) and 349 (0.74%) for ASCVD and no ASCVD, respectively. ^b^A total of 5,924 (9.26%) patients had missing value of BMI: 821 (4.86%) and 5,103 (10.84%) for ASCVD and no ASCVD, respectively. ^c^A total of 28 000 (43.78%) patients had an unspecified stroke subtype: 7,071 (41.84%) and 20,929 (44.47%) in ASCVD and no ASCVD group, respectively. ASCVD, atherosclerotic cardiovascular disease; BMI, body mass index; COPD, chronic obstructive pulmonary disease; IMD, Index of Multiple Deprivation; IQR, interquartile range.

During a median follow-up of 3.6 years (IQR 1.2–6.8), 17,373 patients died without a dementia diagnosis. They were more likely to be older, female, have comorbidities, use health care and receive pre-stroke medications (Supplementary Table 8). The patients transferred out from the CPRD with no dementia diagnosis (*n* = 7,446) shared similar baseline characteristics with those who stayed in the cohort. During follow up, 7,265 patients (11.4%) developed incident dementia, with an incidence rate of 27.3 per 1000 person-years.

Patients with pre-stroke ASCVD had a higher incidence of post-stroke dementia than those without, regardless of age of stroke and gender (Table 2, Supplementary Tables 9 and 10, Supplementary Figure 2), with a crude HR of 1.52 (95% CI: 1.45–1.61) for ASCVD, 1.50 (1.42–1.58) for CHD, and 1.47 (95% CI: 1.34–1.62) for PAD and a minimally adjusted HR of 1.18 (95% CI: 1.12–1.25), 1.16 (95% CI: 1.10–1.23), and 1.25 (95% CI: 1.13–1.37). However, the HRs were progressively reduced in each of our models, and in our full adjustment model the adjusted HR (aHR) was 1.07 (95% CI: 1.00–1.13; *p*-value: 0.04) for ASCVD, 1.04 (95% CI: 0.98–1.11; *p*-value: 0.20) for CHD, and 1.11 (95% CI: 1.00–1.22; *p*-value: 0.05) for PAD (Table 2). No violation of the proportional hazard assumption was found over the 10 years as shown in the log-log plots (Supplementary Figure 3), with a p-value of 0.94, 0.71, and 0.25 for the interaction between time and ASCVD, CHD, and PAD, respectively, in the full adjustment model.

**Table 2:** Association of prior atherosclerotic cardiovascular disease with dementia after stroke

	Prior ASCVD		Prior CHD		Prior PAD	
	Presence	Absence	Presence	Absence	Presence	Absence
	(*n* = 16,900)	(*n* = 47,059)	(*n* = 14,880)	(*n* = 49,079)	(*n* = 3,886)	(*n* = 60,073)
Cases with dementia	2,300	4,965	2,038	5,227	519	6,746
Person-years	60,244	206,127	53,257	213,114	12,876	253,496
Rate ^a^	38.2	24.1	38.3	24.5	40.3	26.6
Crude HR	1.52 (1.45–1.61)	Reference	1.50 (1.42–1.58)	Reference	1.47 (1.34–1.62)	Reference
Adjusted HR
Model 1 ^b^	1.18 (1.12–1.25)	Reference	1.16 (1.10–1.23)	Reference	1.25 (1.13–1.37)	Reference
Model 2 ^c^	1.09 (1.03–1.15)	Reference	1.06 (1.00–1.12)	Reference	1.14 (1.03–1.26)	Reference
Model 3 ^d^	1.07 (1.00–1.13)	Reference	1.04 (0.98–1.11)	Reference	1.11 (1.00–1.22)	Reference

Of 63,959 patients in total, 57,902 patients with complete baseline data were included in all the models for the HR estimates (6,057 were excluded due to missing value in smoking or BMI): 16,046 and 41,856 patients in the ASCVD and no ASCVD group; 14,177 and 43,725 patients in the CHD and no CHD group; 3,651 and 54,251 patients in the PAD and no PAD group, respectively. ^a^Event rates reported in 1,000 person-years calculated from 63,959 patients. ^b^Model 1: adjusted for age (cubic spline variables), gender, IMD, smoking, and BMI (cubic spline variables). ^c^Model 2: adjusted for the variables in model 1 plus comorbidities (stroke subtype, atrial fibrillation, alcohol problem, anxiety, rheumatoid arthritis, asthma, chronic obstructive pulmonary disease, depression, diabetes, epilepsy, hearing loss, heart failure, hyperlipidemia, hypertension, Parkinson’s disease, and transient ischemic attack). The CHD and PAD models additionally adjusted for PAD and CHD, respectively. ^d^Model 3: adjusted for the variables in model 2 plus consultation (cubic spline variables) and medications (statins, other lipid-lowering drugs, anticoagulant, antiplatelet, antihypertensive drugs, and antidiabetic drugs). ASCVD, atherosclerotic cardiovascular disease; BMI, body mass index; CHD, coronary heart disease; HR, hazard ratio; IMD, Index of Multiple Deprivation; PAD, peripheral artery disease.

**Fig. 1:**
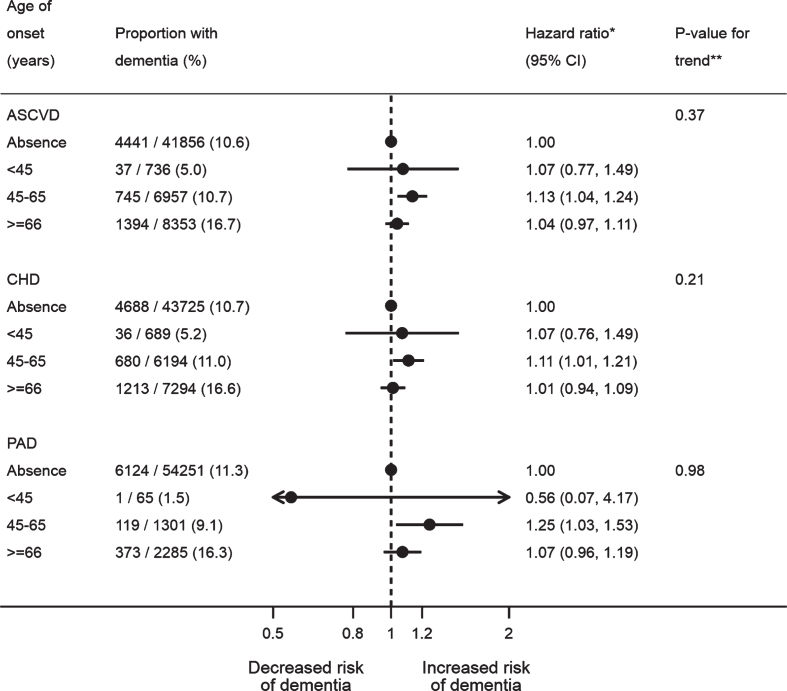
Association between age of atherosclerotic cardiovascular disease onset and dementia after stroke. All the models were adjusted for age, gender, IMD, smoking, BMI, stroke subtype, atrial fibrillation, alcohol problem, anxiety, rheumatoid arthritis, asthma, chronic obstructive pulmonary disease, depression, diabetes, epilepsy, hearing loss, heart failure, hyperlipidemia, hypertension, Parkinson’s disease, transient ischemic attack, consultation, statins, other lipid-lowering drugs, anticoagulant, antiplatelet, antihypertensive drugs, and antidiabetic drugs. The CHD and PAD models additionally adjusted for PAD and CHD, respectively. *A total of 57,902 patients with complete baseline data were included, with the reference group being no prior ASCVD (*n* = 41,856), no prior CHD (*n* = 43,725), or no prior PAD (*n* = 54,251). **Tests for linear trend were conducted in patients with ASCVD/CHD/PAD only, by assigning the medians (ASCVD: 41, 58, and 74; CHD: 41, 58, and 74; PAD: 42, 59, and 75) to the age levels of onset from the lowest to the highest and treating the variable as a numerical variable in the Cox models. ASCVD, atherosclerotic cardiovascular disease; BMI, body mass index; CHD, coronary heart disease; IMD, Index of Multiple Deprivation; PAD, peripheral artery disease.

**Fig. 2:**
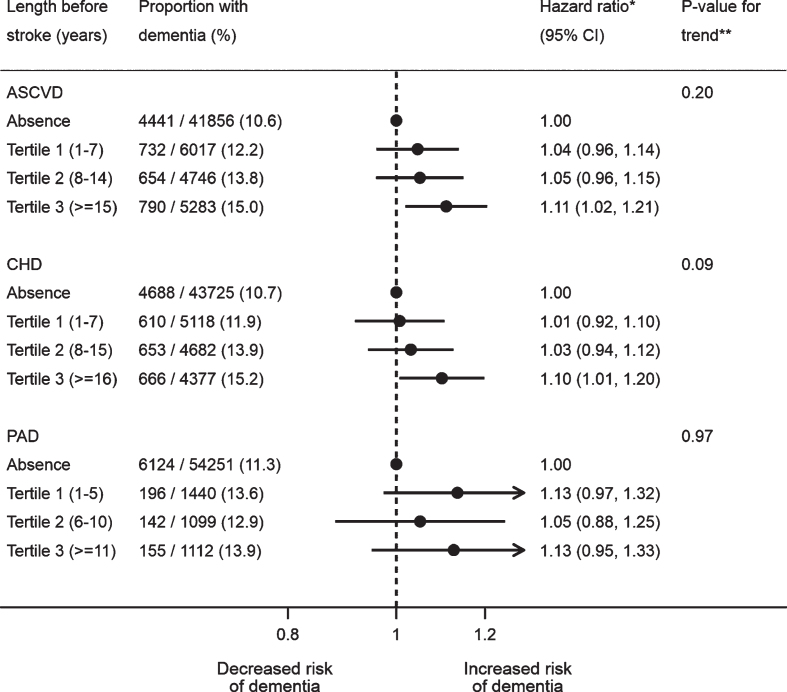
Association between length of time with atherosclerotic cardiovascular disease and dementia after stroke. All the models were adjusted for age, gender, IMD, smoking, BMI, stroke subtype, atrial fibrillation, alcohol problem, anxiety, rheumatoid arthritis, asthma, chronic obstructive pulmonary disease, depression, diabetes, eczema, epilepsy, hyperlipidemia, heart failure, hypertension, irritable bowel syndrome, Parkinson’s disease, transient ischemic attack, consultation, statins, other lipid-lowering drugs, anticoagulant, antiplatelet, antihypertensive drugs, and antidiabetic drugs. The CHD and PAD models additionally adjusted for PAD and CHD, respectively. *A total of 57,902 patients were included, with the reference group being no prior ASCVD (*n* = 41,856), no prior CHD (*n* = 43,725) or no prior PAD (*n* = 54,251). **Tests for linear trend were conducted in patients with ASCVD only, by assigning the medians (ASCVD: 3, 11 and 20; CHD: 3, 11, and 21; PAD: 3, 8, and 15) to the length tertile levels from the lowest to the highest and treating the variable as a numerical variable in the Cox models. ASCVD, atherosclerotic cardiovascular disease; BMI, body mass index; CHD, coronary heart disease; IMD, Index of Multiple Deprivation; PAD, peripheral artery disease.

The crude estimates suggested age of ASCVD onset and the length of time with ASCVD exhibited linear relationships with the risk of post-stroke dementia (Supplementary Tables 11 and 12). Based on the fully adjusted estimates, however, there was no linear trend for the age of ASCVD onset (*p*-value for trend: 0.37 for ASCVD, 0.21 for CHD, and 0.98 for PAD), although mid-life ASCVD (aHR: 1.13; 95% CI: 1.04–1.24; *p*-value: 0.005), CHD (aHR: 1.11; 95% CI: 1.01–1.21; *p*-value: 0.03), and PAD (aHR: 1.25; 95% CI: 1.03–1.53; *p*-value: 0.02) were both associated with dementia after stroke, unlike late life onset (Fig. 1). The adjusted estimates also showed the risk of post-stroke dementia did not increase with the length of time with ASCVD before stroke (*p*-value for trend: 0.20 for ASCVD, 0.09 for CHD, and 0.97 for PAD) (Fig. 2). Neither multiplicative nor additive interaction between CHD and PAD was observed across the adjustment models (all *p*-values for interaction >0.05) (Table 3).

**Table 3:** Interactions between coronary heart disease and peripheral artery disease in the risk of dementia after stroke

	Crude estimate, HR (95% CI)	Adjusted estimate, HR (95% CI)		
		Model 1^a^	Model 2^b^	Model 3^c^
CHD only	1.50 (1.41–1.59)	1.15 (1.09–1.22)	1.06 (1.00–1.13)	1.05 (0.98–1.12)
PAD only	1.53 (1.36–1.73)	1.25 (1.10–1.41)	1.18 (1.04–1.33)	1.13 (1.00–1.28)
CHD plus PAD	1.72 (1.50–1.98)	1.35 (1.18–1.55)	1.17 (1.02–1.35)	1.13 (0.98–1.30)
p-interaction*	0.002	0.51	0.47	0.60
p-interaction**	0.04	0.69	0.51	0.63

Of 63 959 patients in total, 57,902 patients with complete baseline data were included in all the models for the HR estimates (6057 were excluded due to missing value in smoking or BMI). The models included 41,856 patients without any ASCVDs (reference group), 12,395 with CHD alone, 1,869 with PAD alone, and 1,782 with CHD plus PAD. A product term for CHD and PAD was included in all the models. ^a^Model 1: adjusted for age (cubic spline variables), gender, IMD, smoking, and BMI (cubic spline variables). ^b^Model 2: adjusted for the variables in model 1 plus comorbidities (stroke subtype, atrial fibrillation, alcohol problem, anxiety, rheumatoid arthritis, asthma, chronic obstructive pulmonary disease, depression, diabetes, epilepsy, hearing loss, heart failure, hyperlipidemia, hypertension, Parkinson’s disease, and transient ischemic attack. ^c^Model 3: adjusted for the variables in model 2 plus consultation (cubic spline variables) and medications (statins, other lipid-lowering drugs, anticoagulant, antiplatelet, antihypertensive drugs, and antidiabetic drugs). *p-value for testing multiplicative interaction between CHD and PAD. **p-value for testing additive interaction between CHD and PAD. ASCVD, atherosclerotic cardiovascular disease; BMI, body mass index; CHD, coronary heart disease; HR, hazard ratio; IMD, Index of Multiple Deprivation; PAD, peripheral artery disease.

**Fig. 3:**
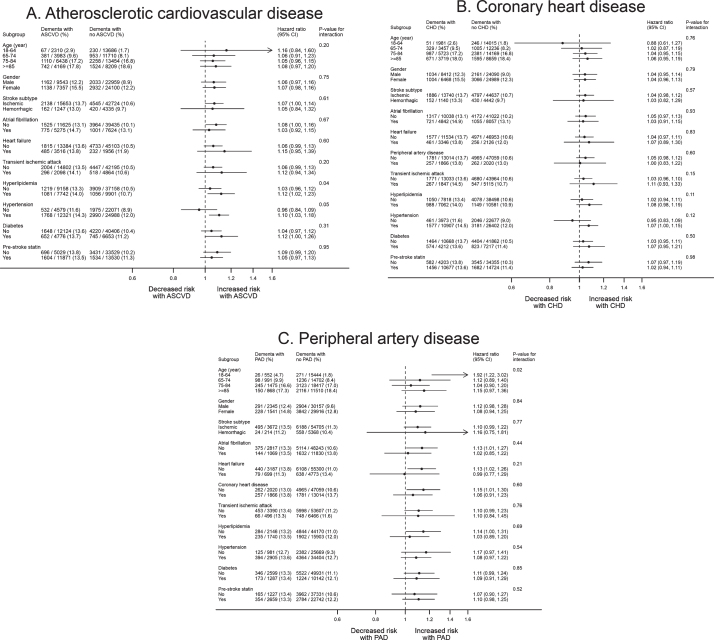
Subgroup analysis for the association of atherosclerotic cardiovascular disease with dementia after stroke. A total of 57,902 patients with complete baseline data were included. All the models were adjusted for age, gender, IMD, smoking, BMI, stroke subtype, atrial fibrillation, alcohol problem, anxiety, rheumatoid arthritis, asthma, chronic obstructive pulmonary disease, depression, diabetes, epilepsy, hyperlipidemia, heart failure, hypertension, Parkinson’s disease, transient ischemic attack, consultation, statins, other lipid-lowering drugs, anticoagulant, antiplatelet, antihypertensive drugs, and antidiabetic drugs. The CHD and PAD models additionally adjusted for PAD and CHD, respectively. ASCVD, atherosclerotic cardiovascular disease; BMI, body mass index; CHD, coronary heart disease; IMD, Index of Multiple Deprivation; PAD, peripheral artery disease.

Our sensitivity analyses did not lead to any important changes to these results (Supplementary Tables 13 to 18). In subgroup analysis (Fig. 3, Supplementary Tables 9 and 10), no patient characteristics were associated with variation in risk of post-stroke dementia in people with prior ASCVD based on the Bonferroni-corrected statistical significance of *p*-value <0.0045.

## DISCUSSION

### Summary of principal findings

In this study, the crude and minimally adjusted estimates (only accounting for demographics and lifestyle) suggested stroke patients with prior ASCVD had a higher risk of dementia. After additional adjustment for multimorbidity and medications, the risk of post-stroke dementia was only slightly increased with ASCVD at a marginal significance level but not significantly associated with pre-existing CHD or PAD. There was no clear evidence that the risk of post-stroke dementia can increase with the age of ASCVD onset or the length of pre-stroke ASCVD, although the risk might be higher among patients with mid-life ASCVD or longer history of ASCVD. Across the adjustment models, we did not find any interactions between CHD and PAD. A series of sensitivity analyses did not change these results appreciably.

### Strengths and limitations

This is the largest patient record investigation to date of the association between prior ASCVD and post-stroke dementia, providing new evidence on how the association may differ by the onset of suffering ASCVD, by the length of pre-existing ASCVD, and by the baseline characteristics. It accounted for more potential confounders and attrition bias than previous studies. The study also benefits from the strengths of the UK’s primary care record system, CPRD, in terms of representativeness of the study population, detailed prescription information, and long duration of follow-up [18].

However, there are limitations associated with CPRD data. Unspecified stroke accounted for 44.0% of stroke patients in our main analysis combined with ischemic stroke. However, this is unlikely to have had an important impact on the results as separating these patients did not change the results. Second, BMI data were missing in 9.3% of patients. Sensitivity analyses assuming extreme values for BMI did not change the results. Third, some important potential predictors of dementia could not be included, such as education, ethnicity, physical activity, and diet, as these data are poorly recorded in the CPRD. Thus, there is the possibility that the modest associations that we observed might still be over-estimates. Furthermore, misclassification in baseline covariates may have resulted in overestimates due to residual confounding. Fourth, there is likely to be under-recording of both ASCVD and dementia in the CPRD [26–28]. It is possible that some patients in the reference groups had underdiagnosed ASCVD. This misclassification would shrink the real association towards the null if these diseases do increase the risk of post-stroke dementia. Conversely, underdiagnosis of dementia or early cognitive impairment might inflate the association as the underdiagnosis was more likely to occur in patients with no ASCVD due to their less contact with health services. In this case, the real association should be closer to the null than our estimates. Finally, the statistical power may not be sufficient to detect significant difference for some of the interaction, trend and subgroup analyses.

### Comparisons with other studies

While our crude and minimally adjusted estimates suggested a positive association of prior CHD and PAD with dementia after stroke, the association was not found based on the fully adjusted estimates. These findings are consistent with a recent systematic review [13]. It included 15 cohort studies and found a positive association between CHD and post-stroke dementia, with a pooled crude odds ratio (OR) of 1.32 (95% CI: 1.11–1.58) [13]. Only two studies included in this review report adjusted estimates for CHD (pooled HR: 1.11; 95% CI: 0.85–1.44) [14, 15]. Evidence focusing on post-stroke dementia associated with PAD is very limited, with two studies contributing to a pooled crude OR of 3.59 (95% CI: 1.47–8.76) [29, 30] and only one study reporting an adjusted estimate (HR: 1.27; 95% CI: 0.90–1.79) [15]. The smaller estimates in our study probably reflect that there was less attrition bias and more adjustment for confounding than in the previous studies.

Compared with the findings of other systematic reviews from the general population (the pooled adjusted estimates were 1.26 (95% CI: 1.06–1.49) [7] and 1.45 (95% CI: 1.21–1.74) [8] for CHD and 1.50 (95% CI: 1.10–1.45) [9] for PAD), our study showed that the association with dementia was weaker for both CHD and PAD among stroke patients. These results suggest that stroke may lie on the causal pathway between atherosclerosis and dementia or that the direct impact of atherosclerosis on the occurrence of dementia among stroke patients may be different from that of the general population. Another possible reason for the weaker association is that deaths after stroke may be more common in those most vulnerable to dementia; however, this is not supported by our competing-risks analysis treating death as a competing-risks event (Supplementary Tables 17 and 18), which further pulled the estimates towards the null.

There has been a large body of literature linking dementia to a variety of mid-life vascular factors shared by ASCVD, such as blood cholesterol, blood pressure, blood glucose, and overweight/obesity [31, 32]. However, our study did not find evidence that the risk of post-stroke dementia in patients with mid-life ASCVD was significantly higher than that in patients with earlier or later onset of the disease. Although our study showed mid-life ASCVD and longer history of ASCVD were associated with an increased risk of dementia, whether the risk can be varied by the age of ASCVD onset or the length of ASCVD history requires further studies to confirm.

### Implications for practice

The raised risk suggests that prior ASCVD could be used as a marker for clinicians to consider more monitoring of cognitive function post-stroke in this population subgroup. This risk is independent of age, gender, socioeconomic status, and lifestyle, but is at least partly due to comorbidity. After additional adjustment for pre-stroke medications, the risk was only decreased by a small degree, suggesting these medications in patients with prior ASCVD may have limited protective effects on cognitive function following stroke. Given prior CHD/PAD (markers of atherosclerosis) is not independently associated with increased risk of post-stroke dementia beyond that associated with stroke itself, the potential effectiveness of statins in preventing post-stroke cognitive decline observed in previous studies may be due to their effect on reducing risk of recurrent stroke and other pleiotropic effects [13, 33].

### Implications for future research

Our results highlight the importance of adequately adjusting for known risk factors for dementia when investigating the relationship between atherosclerosis (and possibly other cardiovascular risk factors) and dementia in stroke patients. Further research would help clarify whether the risk of post-stroke dementia depends on the timing and duration of prior atherosclerosis. Randomized controlled trials and real-world evidence are needed to determine targeted interventions to reduce the risk of dementia among stroke patients with prior atherosclerosis.

### Conclusions

Stroke patients with prior ASCVD are more likely to develop subsequent dementia. After full adjustment for potential baseline confounding, however, the risk of post-stroke dementia is attenuated with only a slight increase with prior ASCVD. It is important to adequately account for known risk factors for dementia when investigating the association of cardiovascular risk factors with dementia in stroke patients. The possibility that risk of post-stroke dementia might vary by age of ASCVD onset and length of ASCVD history could be explored in further studies.

## Supplementary Material

Supplementary Material
