# Supplementary material for: Association of Prior Atherosclerotic Cardiovascular Disease with Dementia After Stroke: A Retrospective Cohort Study
Source: J Alzheimers Dis. 2020 Sep 29;77(3):1157–67. doi: 10.3233/JAD-200536 (PMC7683071; doi:10.3233/JAD-200536)
Supplement: Supplementary Material [file jad-77-jad200536-s001.pdf]

Supplementary Material

Association of Prior Atherosclerotic Cardiovascular Disease with Dementia After Stroke: A Retrospective Cohort Study

		Page
Supplementary Table 1	Codes for stroke	S1
Supplementary Table 2	Codes for dementia	S3
Supplementary Table 3	Codes for coronary heart disease	S7
Supplementary Table 4	Codes for peripheral artery disease	S14
Supplementary Table 5	Criteria for quality control	S15
Supplementary Table 6	Baseline characteristics by coronary heart disease or peripheral artery disease	S16
Supplementary Table 7	Baseline characteristics of patients with complete and incomplete baseline data	S17
Supplementary Table 8	Baseline characteristics stratified according to follow-up status	S18
Supplementary Table 9	Association of prior atherosclerotic cardiovascular disease with dementia after stroke: stratified by age of stroke	S19
Supplementary Table 10	Association of prior atherosclerotic cardiovascular disease with dementia after stroke: stratified by gender	S21
Supplementary Table 11	Crude and partially adjusted association between the age of prior atherosclerotic cardiovascular disease onset and dementia after stroke	S22
Supplementary Table 12	Crude and partially adjusted association between length of time with prior atherosclerotic cardiovascular disease and dementia after stroke	S23
Supplementary Table 13	Sensitivity analysis excluding patients having a first record of dementia within the first 6-month follow-up	S24
Supplementary Table 14	Sensitivity analysis separating unspecified stroke from ischemic stroke	S25
Supplementary Table 15	Sensitivity analysis restricting to patients with linkage to Hospital Episode Statistics	S26
Supplementary Table 16	Sensitivity analysis changing missing BMI to 5th or 95th percentile and changing missing smoking to never or current smoking	S27
Supplementary Table 17	Sensitivity analysis using competing-risks regression models	S29
Supplementary Table 18	A series of sensitivity analyses on the interaction between coronary heart disease and peripheral artery disease in the risk of dementia after stroke	S30
Supplementary Figure 1	Flow chart of patient inclusion	S32
Supplementary Figure 2	Kaplan-Meier plots	S33
Supplementary Figure 3	Log-log plots	S34
Checklist	The RECORD statement – checklist of items, extended from the STROBE statement	S35

Supplementary Table 1. Codes for stroke

Read codes used in CPRD

Medcode	Readcode	Description
569	G64..12	Infarction - cerebral
1298	G66..11	CVA unspecified
1469	G66..00	Stroke and cerebrovascular accident unspecified
2418	G6...00	Cerebrovascular disease
3149	G64z.00	Cerebral infarction NOS
3535	G61z.00	Intracerebral hemorrhage NOS
5051	G61..00	Intracerebral hemorrhage
5185	G64z111	Lateral medullary syndrome
5363	G64..11	CVA - cerebral artery occlusion
5602	G64z.12	Cerebellar infarction
6116	G66..13	CVA - Cerebrovascular accident unspecified
6155	G64..13	Stroke due to cerebral arterial occlusion
6253	G66..12	Stroke unspecified
6960	G61..11	CVA - cerebrovascular acid due to intracerebral hemorrhage
7780	G667.00	Left sided CVA
7912	G614.00	Pontine hemorrhage
8443	G663.00	Brain stem stroke syndrome
8837	G64..00	Cerebral arterial occlusion
9985	G64z200	Left sided cerebral infarction
10504	G64z300	Right sided cerebral infarction
12833	G668.00	Right sided CVA
13564	G613.00	Cerebellar hemorrhage
15019	G641.00	Cerebral embolism
15252	G64z.11	Brainstem infarction NOS
16517	G640.00	Cerebral thrombosis
16956	G669.00	Cerebral palsy, not congenital or infantile, acute
17322	G664.00	Cerebellar stroke syndrome
18604	G61..12	Stroke due to intracerebral hemorrhage
18689	G660.00	Middle cerebral artery syndrome
19201	G61X100	Right sided intracerebral hemorrhage, unspecified
19260	G662.00	Posterior cerebral artery syndrome
19280	G661.00	Anterior cerebral artery syndrome
23671	G63y000	Cerebral infarct due to thrombosis of precerebral arteries
24446	G63y100	Cerebral infarction due to embolism of precerebral arteries
25615	G64z000	Brainstem infarction
26424	G64z400	Infarction of basal ganglia
27975	G641000	Cerebral infarction due to embolism of cerebral arteries
28314	G61X000	Left sided intracerebral hemorrhage, unspecified
30045	G616.00	External capsule hemorrhage

30202	G617.00	Intracerebral hemorrhage, intraventricular
31060	G61X.00	Intracerebral hemorrhage in hemisphere, unspecified
31595	G610.00	Cortical hemorrhage
33499	G665.00	Pure motor lacunar syndrome
33543	G6X..00	Cerebral infarct due/unspcf occlusn or sten/cerebrl artrs
34758	G641.11	Cerebral embolus
36717	G640000	Cerebral infarction due to thrombosis of cerebral arteries
39344	G676000	Cereb infarct due cerebral venous thrombosis, nonpyogenic
40338	G611.00	Internal capsule hemorrhage
40758	G6W..00	Cereb infarct due unsp occlus/stenos precerebr arteries
44765	G653.00	Carotid artery syndrome hemispheric
46316	G612.00	Basal nucleus hemorrhage
47642	G64z100	Wallenberg syndrome
50594	G654.00	Multiple and bilateral precerebral artery syndromes
51767	G666.00	Pure sensory lacunar syndrome
53745	Gyu6400	[X]Other cerebral infarction
55247	G65z000	Impending cerebral ischaemia
57315	G618.00	Intracerebral hemorrhage, multiple localized
62342	G615.00	Bulbar hemorrhage
90572	Gyu6500	[X]Occlusion and stenosis of other precerebral arteries
91627	Gyu6300	[X]Cerebrl infarctn due/unspcf occlusn or sten/cerebrl artrs
92036	Gyu6600	[X]Occlusion and stenosis of other cerebral arteries
94482	Gyu6G00	[X]Cereb infarct due unsp occlus/stenos precerebr arteries
96630	Gyu6F00	[X]Intracerebral hemorrhage in hemisphere, unspecified

ICD-10 Codes used in HES and ONS

ICD Code	Description
I63	Cerebral infarction
I63.0	Cerebral infarction due to thrombosis of precerebral arteries
I63.1	Cerebral infarction due to embolism of precerebral arteries
I63.2	Cerebral infarction due to unspecified occlusion or stenosis of precerebral arteries
I63.3	Cerebral infarction due to thrombosis of cerebral arteries
I63.4	Cerebral infarction due to embolism of cerebral arteries
I63.5	Cerebral infarction due to unspecified occlusion or stenosis of cerebral arteries
I63.6	Cerebral infarction due to cerebral venous thrombosis, nonpyogenic
I63.8	Other cerebral infarction
I63.9	Cerebral infarction, unspecified
I64	Stroke, not specified as hemorrhage or infarction
I64.0	Stroke, not specified as hemorrhage or infarction
I61	Intracerebral hemorrhage
I61.0	Intracerebral hemorrhage in hemisphere, subcortical
I61.1	Intracerebral hemorrhage in hemisphere, cortical
I61.2	Intracerebral hemorrhage in hemisphere, unspecified

I61.3	Intracerebral hemorrhage in brain stem
I61.4	Intracerebral hemorrhage in cerebellum
I61.5	Intracerebral hemorrhage, intraventricular
I61.6	Intracerebral hemorrhage, multiple localized
I61.8	Other intracerebral hemorrhage
I61.9	Intracerebral hemorrhage, unspecified

Supplementary Table 2. Codes for dementia

Read codes used in CPRD

Medcode	Readcode	Description
1350	E00..12	Senile/presenile dementia
1916	E00..11	Senile dementia
1917	F110.00	Alzheimer's disease
4357	Eu02z14	[X] Senile dementia NOS
4693	Eu02z00	[X] Unspecified dementia
5931	1461.00	H/O: dementia
6578	Eu01.00	[X]Vascular dementia
7323	E000.00	Uncomplicated senile dementia
7572	F116.00	Lewy body disease
7664	Eu00.00	[X]Dementia in Alzheimer's disease
8195	Eu00z11	[X]Alzheimer's dementia unspec
8634	E004.11	Multi infarct dementia
8934	Eu01200	[X]Subcortical vascular dementia
9509	Eu02300	[X]Dementia in Parkinson's disease
9565	Eu01.11	[X]Arteriosclerotic dementia
11136	F111.00	Pick's disease
11175	Eu01100	[X]Multi-infarct dementia
11379	Eu00112	[X]Senile dementia, Alzheimer's type
12621	Eu02.00	[X]Dementia in other diseases classified elsewhere
15165	E001.00	Presenile dementia
16797	F110000	Alzheimer's disease with early onset
18386	E002000	Senile dementia with paranoia
19393	Eu01z00	[X]Vascular dementia, unspecified
19477	E004.00	Arteriosclerotic dementia
21887	E002100	Senile dementia with depression
25386	E041.00	Dementia in conditions EC
25704	Eu00011	[X]Presenile dementia, Alzheimer's type
26270	Eu02500	[X]Lewy body dementia
26323	Eu10711	[X]Alcoholic dementia NOS
27342	E012.11	Alcoholic dementia NOS
27677	E001300	Presenile dementia with depression
27759	Eu02z16	[X] Senile dementia, depressed or paranoid type
28402	Eu02000	[X]Dementia in Pick's disease
29386	Eu00z00	[X]Dementia in Alzheimer's disease, unspecified
30032	E001200	Presenile dementia with paranoia
30706	Eu00200	[X]Dementia in Alzheimer's dis, atypical or mixed type
31016	Eu01300	[X]Mixed cortical and subcortical vascular dementia
32057	F110100	Alzheimer's disease with late onset
33707	E00..00	Senile and presenile organic psychotic conditions

34944	Eu02z13	[X] Primary degenerative dementia NOS
37014	Eu02200	[X]Dementia in Huntington's disease
37015	E003.00	Senile dementia with delirium
38438	E001z00	Presenile dementia NOS
38678	Eu00100	[X]Dementia in Alzheimer's disease with late onset
41089	E002z00	Senile dementia with depressive or paranoid features NOS
41185	Eu02400	[X]Dementia in human immunodef virus [HIV] disease
42279	E004z00	Arteriosclerotic dementia NOS
42602	E001000	Uncomplicated presenile dementia
43089	E004000	Uncomplicated arteriosclerotic dementia
43292	E004300	Arteriosclerotic dementia with depression
43346	Eu00113	[X]Primary degen dementia of Alzheimer's type, senile onset
44674	E002.00	Senile dementia with depressive or paranoid features
46488	Eu01000	[X]Vascular dementia of acute onset
46762	Eu00111	[X]Alzheimer's disease type 1
48501	Eu02z11	[X] Presenile dementia NOS
49263	Eu00000	[X]Dementia in Alzheimer's disease with early onset
49513	E001100	Presenile dementia with delirium
53446	Eu04100	[X]Delirium superimposed on dementia
54106	Eu02100	[X]Dementia in Creutzfeldt-Jakob disease
54505	E012.00	Other alcoholic dementia
55313	Eu01y00	[X]Other vascular dementia
55467	E004200	Arteriosclerotic dementia with paranoia
55838	Eu01111	[X]Predominantly cortical dementia
56912	E004100	Arteriosclerotic dementia with delirium
59122	Fyu3000	[X]Other Alzheimer's disease
60059	Eu00012	[X]Primary degen dementia, Alzheimer's type, presenile onset
61528	Eu00013	[X]Alzheimer's disease type 2
64267	Eu02y00	[X]Dementia in other specified diseases class if elsewhere

ICD-10 Codes used in HES and ONS

ICD Code	Description
F00	Dementia in Alzheimer disease
F00.0	Dementia in Alzheimer disease with early onset
F00.1	Dementia in Alzheimer disease with late onset
F00.2	Dementia in Alzheimer disease, atypical or mixed type
F00.9	Dementia in Alzheimer disease, unspecified
F01	Vascular dementia
F01.0	Vascular dementia of acute onset
F01.1	Multi-infarct dementia
F01.2	Subcortical vascular dementia
F01.3	Mixed cortical and subcortical vascular dementia
F01.8	Other vascular dementia

F01.9	Vascular dementia, unspecified
F02	Dementia in other diseases classified elsewhere
F02.0	Dementia in Pick disease
F02.1	Dementia in Creutzfeldt-Jakob disease
F02.2	Dementia in Huntington disease
F02.3	Dementia in Parkinson disease
F02.4	Dementia in human immunodeficiency virus [HIV] disease
F02.8	Dementia in other specified diseases classified elsewhere
F03	Unspecified dementia
F05.1	Delirium superimposed on dementia
G30	Alzheimer disease
G30.0	Alzheimer disease with early onset
G30.1	Alzheimer disease with late onset
G30.8	Other Alzheimer disease
G30.9	Alzheimer disease, unspecified
G31.0	Circumscribed brain atrophy
G31.8	Other specified degenerative diseases of nervous system

Supplementary Table 3. Codes for coronary heart disease

Read codes used in CPRD

Medcode	Readcode	Description
240	G3...00	Ischemic heart disease
241	G30..00	Acute myocardial infarction
732	7928z00	Transluminal balloon angioplasty of coronary artery NOS
737	792..11	Coronary artery bypass graft operations
1204	G30..14	Heart attack
1344	G340.12	Coronary artery disease
1414	G33z300	Angina on effort
1430	G33..00	Angina pectoris
1431	G311.13	Unstable angina
1655	G340.11	Triple vessel disease of the heart
1676	G3z..00	Ischemic heart disease NOS
1677	G30..15	MI - acute myocardial infarction
1678	G308.00	Inferior myocardial infarction NOS
1792	G3...13	IHD - Ischemic heart disease
2155	G341000	Ventricular cardiac aneurysm
2491	G30..12	Coronary thrombosis
2901	7928	Transluminal balloon angioplasty of coronary artery
3159	792Dy00	Other specified other bypass of coronary artery
3704	G307.00	Acute subendocardial infarction
3999	G340000	Single coronary vessel disease
4017	G32..00	Old myocardial infarction
4656	G311.11	Crescendo angina
5030	ZV45K00	[V]Presence of coronary artery bypass graft
5254	G340100	Double coronary vessel disease
5387	G301.00	Other specified anterior myocardial infarction
5413	G340.00	Coronary atherosclerosis
5674	ZV45K11	[V]Presence of coronary artery bypass graft - CABG
5703	7928.11	Percutaneous balloon coronary angioplasty
5744	7927500	Open angioplasty of coronary artery
5904	792..00	Coronary artery operations
6182	7929y00	Other therapeutic transluminal op on coronary artery OS
6331	G341.00	Aneurysm of heart
6336	14A5.00	H/O: angina pectoris
6980	ZV45L00	[V]Status following coronary angioplasty NOS
7134	7921.11	Other autograft bypass of coronary artery
7137	7920y00	Saphenous vein graft replacement of coronary artery OS
7320	G343.00	Ischemic cardiomyopathy
7347	G311100	Unstable angina
7442	7920200	Saphenous vein graft replacement of three coronary arteries

7609	7921z00	Other autograft replacement of coronary artery NOS
7634	7920100	Saphenous vein graft replacement of two coronary arteries
7696	G33z200	Syncope anginosa
8312	7920.11	Saphenous vein graft bypass of coronary artery
8568	G37..00	Cardiac syndrome X
8679	7920000	Saphenous vein graft replacement of one coronary artery
8935	G302.00	Acute inferolateral infarction
8942	7929400	Insertion of coronary artery stent
9276	G31y000	Acute coronary insufficiency
9413	G31y.00	Other acute and subacute ischemic heart disease
9414	7921	Other autograft replacement of coronary artery
9507	G307000	Acute non-Q wave infarction
9555	G33z500	Post infarct angina
10209	7921200	Autograft replacement of three coronary arteries NEC
10562	G307100	Acute non-ST segment elevation myocardial infarction
10603	792z.00	Coronary artery operations NOS
11048	G331.11	Variant angina pectoris
11610	7920300	Saphenous vein graft replacement of four+ coronary arteries
11983	G311500	Acute coronary syndrome
12139	G300.00	Acute anterolateral infarction
12229	G30X000	Acute ST segment elevation myocardial infarction
12734	SP07600	Coronary artery bypass graft occlusion
12804	G33z700	Stable angina
12986	G331.00	Prinzmetal's angina
13566	G30..11	Attack - heart
13571	G30..16	Thrombosis - coronary
14658	G30z.00	Acute myocardial infarction NOS
14897	G301z00	Anterior myocardial infarction NOS
14898	G305.00	Lateral myocardial infarction NOS
15661	G310.11	Dressler's syndrome
15754	G34z.00	Other chronic ischemic heart disease NOS
16408	G32..11	Healed myocardial infarction
17133	G30A.00	Mural thrombosis
17307	G311200	Angina at rest
17464	G32..12	Personal history of myocardial infarction
17689	G30..17	Silent myocardial infarction
17872	G301100	Acute anteroseptal infarction
18118	G311400	Worsening angina
18125	G330000	Nocturnal angina
18249	7920	Saphenous vein graft replacement of coronary artery
18643	ZV45800	[V]Presence of coronary angioplasty implant and graft
18670	7928000	Percut transluminal balloon angioplasty one coronary artery
18842	G35..00	Subsequent myocardial infarction

18889	G34z000	Asymptomatic coronary heart disease
18913	ZV45700	[V]Presence of aortocoronary bypass graft
19046	7929300	Rotary blade coronary angioplasty
19193	7923z00	Prosthetic replacement of coronary artery NOS
19402	7923	Prosthetic replacement of coronary artery
19413	7921100	Autograft replacement of two coronary arteries NEC
19655	G311.14	Angina at rest
20095	G330.00	Angina decubitus
20903	7A6G100	Peroperative angioplasty
21844	G31y300	Transient myocardial ischaemia
22020	792B000	Endarterectomy of coronary artery NEC
22383	G3y..00	Other specified ischemic heart disease
22647	7925311	LIMA single anastomosis
22828	7929000	Percutaneous transluminal laser coronary angioplasty
23078	G34y100	Chronic myocardial ischaemia
23579	G310.00	Postmyocardial infarction syndrome
23708	G361.00	Atrial septal defect/curr comp folow acut myocardal infarct
23892	G304.00	Posterior myocardial infarction NOS
24126	G360.00	Haemopericardium/current comp folow acut myocard infarct
24540	G34y000	Chronic coronary insufficiency
24888	7929	Other therapeutic transluminal operations on coronary artery
25842	G33z.00	Angina pectoris NOS
26863	G33z600	New onset angina
27484	G341.11	Cardiac aneurysm
27951	G31..00	Other acute and subacute ischemic heart disease
27977	G31yz00	Other acute and subacute ischemic heart disease NOS
28138	G34..00	Other chronic ischemic heart disease
28554	G33zz00	Angina pectoris NOS
28736	G30y000	Acute atrial infarction
28837	7925.11	Creation of bypass from mammary artery to coronary artery
29421	G344.00	Silent myocardial ischaemia
29553	G366.00	Thrombosis atrium,auric append&vent/curr comp foll acute MI
29643	G303.00	Acute inferoposterior infarction
29758	G30X.00	Acute transmural myocardial infarction of unspecif site
29902	G330z00	Angina decubitus NOS
30330	G309.00	Acute Q-wave infarct
30421	G30..13	Cardiac rupture following myocardial infarction (MI)
31519	7925100	Double implant of mammary arteries into coronary arteries
31540	7924200	Revision of bypass for three coronary arteries
31556	7922	Allograft replacement of coronary artery
31571	792y.00	Other specified operations on coronary artery
31679	7929z00	Other therapeutic transluminal op on coronary artery NOS
32272	G38..00	Postoperative myocardial infarction

32450	G33z400	Ischemic chest pain
32651	7922.11	Allograft bypass of coronary artery
32854	G30B.00	Acute posterolateral myocardial infarction
33461	7924	Revision of bypass for coronary artery
33471	792Dz00	Other bypass of coronary artery NOS
33620	792B.00	Repair of coronary artery NEC
33650	7929100	Percut transluminal coronary thrombolysis with streptokinase
33718	7925000	Double anastomosis of mammary arteries to coronary arteries
33735	7928100	Percut translum balloon angioplasty mult coronary arteries
34328	G311300	Refractory angina
34633	G34y.00	Other specified chronic ischemic heart disease
34803	G30y.00	Other acute myocardial infarction
34963	792D.00	Other bypass of coronary artery
34965	792A.00	Diagnostic transluminal operations on coronary artery
35119	G501.00	Post infarction pericarditis
35674	14A3.00	H/O: myocardial infarct <60
35713	G34yz00	Other specified chronic ischemic heart disease NOS
36011	7923.11	Prosthetic bypass of coronary artery
36423	G36..00	Certain current complication follow acute myocardial infarct
36523	G311.00	Preinfarction syndrome
36609	G342.00	Atherosclerotic cardiovascular disease
36854	G332.00	Coronary artery spasm
37657	G362.00	Ventric septal defect/curr comp fol acut myocardal infarctn
37682	7925	Connection of mammary artery to coronary artery
37719	7925y00	Connection of mammary artery to coronary artery OS
38609	G351.00	Subsequent myocardial infarction of inferior wall
38813	7A54500	Rotary blade angioplasty
39449	G312.00	Coronary thrombosis not resulting in myocardial infarction
39546	Gyu3000	[X]Other forms of angina pectoris
39655	G311.12	Impending infarction
39693	G31y200	Subendocardial ischaemia
40399	14A4.00	H/O: myocardial infarct >60
40429	G301000	Acute anteroapical infarction
40996	7929111	Percut translum coronary thrombolytic therapy- streptokinase
41221	G30y200	Acute septal infarction
41547	7928y00	Transluminal balloon angioplasty of coronary artery OS
41677	G341z00	Aneurysm of heart NOS
41757	7927z00	Other open operation on coronary artery NOS
41835	G384.00	Postoperative subendocardial myocardial infarction
42304	7929500	Insertion of drug-eluting coronary artery stent
42462	7928200	Percut translum balloon angioplasty bypass graft coronary a
42708	7921300	Autograft replacement of four of more coronary arteries NEC
43939	793G.00	Perc translumin balloon angioplasty stenting coronary artery

44561	7921000	Autograft replacement of one coronary artery NEC
44585	792Bz00	Repair of coronary artery NOS
44723	7925200	Single anast mammary art to left ant descend coronary art
45370	7922300	Allograft replacement of four or more coronary arteries
45809	G350.00	Subsequent myocardial infarction of anterior wall
45886	7922200	Allograft replacement of three coronary arteries
46017	G30yz00	Other acute myocardial infarction NOS
46112	G380.00	Postoperative transmural myocardial infarction anterior wall
46166	G35X.00	Subsequent myocardial infarction of unspecified site
46276	G381.00	Postoperative transmural myocardial infarction inferior wall
47788	7927	Other open operations on coronary artery
48206	7927300	Transposition of coronary artery NEC
48767	7922z00	Allograft replacement of coronary artery NOS
48822	7925011	LIMA sequential anastomosis
50372	14AH.00	H/O: Myocardial infarction in last year
51507	7925300	Single anastomosis of mammary artery to coronary artery NEC
51515	7920z00	Saphenous vein graft replacement coronary artery NOS
51702	7927400	Exploration of coronary artery
52938	7924000	Revision of bypass for one coronary artery
54251	G311z00	Preinfarction syndrome NOS
54535	G33z100	Stenocardia
55092	792C000	Replacement of coronary arteries using multiple methods
55137	G311011	MI - myocardial infarction aborted
55598	792C.00	Other replacement of coronary artery
56905	792Ay00	Diagnostic transluminal operation on coronary artery OS
56990	7925z00	Connection of mammary artery to coronary artery NOS
57062	14AJ.00	H/O: Angina in last year
57241	7922100	Allograft replacement of two coronary arteries
57634	7924z00	Revision of bypass for coronary artery NOS
59189	G363.00	Ruptur cardiac wall w'out haemopericard/cur comp fol ac MI
59193	G341200	Aneurysm of coronary vessels
59423	7922y00	Other specified allograft replacement of coronary artery
59940	G364.00	Ruptur chordae tendinae/curr comp fol acute myocard infarct
60067	793G000	Perc translum ball angio insert 1-2 drug elut stents cor art
60753	7926300	Single implantation thoracic artery into coronary artery NEC
61208	793Gz00	Perc translum balloon angioplasty stenting coronary art NOS
61248	792Az00	Diagnostic transluminal operation on coronary artery NOS
61310	7921y00	Other autograft replacement of coronary artery OS
62608	7926000	Double anastom thoracic arteries to coronary arteries NEC
62626	G30y100	Acute papillary muscle infarction
63153	7924500	Revision of implantation of thoracic artery into heart
63467	G306.00	True posterior myocardial infarction
64923	7A6H300	Prosthetic graft patch angioplasty

66236	7923200	Prosthetic replacement of three coronary arteries
66388	G33z000	Status anginosus
66583	7929200	Percut translum inject therap subst to coronary artery NEC
66664	7923100	Prosthetic replacement of two coronary arteries
66921	7A6H400	Percutaneous transluminal angioplasty of vascular graft
67087	G341100	Other cardiac wall aneurysm
67554	7924100	Revision of bypass for two coronary arteries
67591	7926200	Single anastomosis of thoracic artery to coronary artery NEC
67761	7923300	Prosthetic replacement of four or more coronary arteries
68123	7925312	RIMA single anastomosis
68139	7925400	Single implantation of mammary artery into coronary artery
68357	G31y100	Microinfarction of heart
68748	G38z.00	Postoperative myocardial infarction, unspecified
69247	792By00	Other specified repair of coronary artery
69474	G365.00	Rupture papillary muscle/curr comp fol acute myocard infarct
70111	7922000	Allograft replacement of one coronary artery
70755	792Cz00	Replacement of coronary artery NOS
72562	G353.00	Subsequent myocardial infarction of other sites
72780	7926z00	Connection of other thoracic artery to coronary artery NOS
85947	793G200	Perc translum balloon angioplasty insert 1-2 stents cor art
86071	7928300	Percut translum cutting balloon angioplasty coronary artery
87849	793G100	Perc tran ball angio ins 3 or more drug elut stents cor art
91774	G341300	Acquired atrioventricular fistula of heart
92233	7925012	RIMA sequential anastomosis
92419	7923000	Prosthetic replacement of one coronary artery
92927	793G300	Percutaneous cor balloon angiop 3 more stents cor art NEC
93618	7929600	Percutaneous transluminal atherectomy of coronary artery
93706	793H000	Percutaneous transluminal balloon dilation cardiac conduit
93828	792Cy00	Other specified replacement of coronary artery
95382	7927y00	Other specified other open operation on coronary artery
96804	7926	Connection of other thoracic artery to coronary artery
96838	Gyu3400	[X]Acute transmural myocardial infarction of unspecif site
97953	7924y00	Other specified revision of bypass for coronary artery
99991	Gyu3600	[X]Subsequent myocardial infarction of unspecified site
101569	7924300	Revision of bypass for four or more coronary arteries
105250	G341111	Mural cardiac aneurysm
105479	G39..00	Coronary microvascular disease
106812	G383.00	Postoperative transmural myocardial infarction unspec site
109035	Gyu3500	[X]Subsequent myocardial infarction of other sites

ICD-10 Codes used in HES and ONS

ICD Code	Description
I20	Angina pectoris

I20.0	Unstable angina
I20.1	Angina pectoris with documented spasm
I20.8	Other forms of angina pectoris
I20.9	Angina pectoris, unspecified
I21	Acute myocardial infarction
I21.0	Acute transmural myocardial infarction of anterior wall
I21.1	Acute transmural myocardial infarction of inferior wall
I21.2	Acute transmural myocardial infarction of other sites
I21.3	Acute transmural myocardial infarction of unspecified site
I21.4	Acute subendocardial myocardial infarction
I21.9	Acute myocardial infarction, unspecified
I22	Subsequent myocardial infarction
I22.0	Subsequent myocardial infarction of anterior wall
I22.1	Subsequent myocardial infarction of inferior wall
I22.8	Subsequent myocardial infarction of other sites
I22.9	Subsequent myocardial infarction of unspecified site
I23	Certain current complications following acute myocardial infarction
I23.0	Haemopericardium as current complication following acute myocardial infarction
I23.1	Atrial septal defect as current complication following acute myocardial infarction
I23.2	Ventricular septal defect as current complication following acute myocardial infarction
I23.3	Rupture of cardiac wall without haemopericardium as current complication following acute myocardial infarction
I23.4	Rupture of chordae tendineae as current complication following acute myocardial infarction
I23.5	Rupture of papillary muscle as current complication following acute myocardial infarction
I23.6	Thrombosis of atrium, auricular appendage, and ventricle as current complications following acute myocardial infarction
I23.8	Other current complications following acute myocardial infarction
I24	Other acute ischemic heart diseases
I24.0	Coronary thrombosis not resulting in myocardial infarction
I24.1	Dressler syndrome
I24.8	Other forms of acute ischemic heart disease
I24.9	Acute ischemic heart disease, unspecified
I25	Chronic ischemic heart disease
I25.0	Atherosclerotic cardiovascular disease, so described
I25.1	Atherosclerotic heart disease
I25.2	Old myocardial infarction
I25.3	Aneurysm of heart
I25.4	Coronary artery aneurysm and dissection
I25.5	Ischemic cardiomyopathy
I25.6	Silent myocardial ischaemia
I25.8	Other forms of chronic ischemic heart disease
I25.9	Chronic ischemic heart disease, unspecified

Supplementary Table 4. Codes for peripheral artery disease

Read codes used in CPRD

Medcode	Readcode	Description
1517	G73z000	Intermittent claudication
2760	G73zz00	Peripheral vascular disease NOS
3530	G73z.00	Peripheral vascular disease NOS
5943	G73..00	Other peripheral vascular disease
18499	662U.00	Peripheral vascular disease monitoring

ICD-10 Codes used in HES and ONS

ICD Code	Description
I73.9	Peripheral vascular disease, unspecified

Supplementary Table 5. Criteria for quality control

Data item	Unacceptable value
**ALL the records of a patient were excluded for any reason below:**	
First registration date	Empty; invalid date; prior to year of birth; within one year before the first stroke diagnosis date
Current registration date	Invalid date; prior to first registration date; prior to year of birth
Transferred out date	Invalid date; present with no reason; prior to first registration date; prior to current registration date
A transferred out reason	Present with no date
Registration status	Temporary patients
Age	Over 125 years at the end of follow-up
Year of birth	Absent
Gender	Other than male, female or indeterminate
Death date	Prior to the first registration date; prior to the current registration date
**RELEVANT episode records of a patient were excluded for any reason below:**	
Event date	Invalid; absent; prior to birth year
Weight	<30kg; >300kg
Height	<1.1 metres; >2.3 metres
**The date were CHANGED for any reason below:**	
Change the death date and transferred out date to the first stroke diagnosis date	Death date prior to the first stroke diagnosis date; transferred out date prior to the first stroke diagnosis date
Change the death date and transferred out date to the first date of dementia diagnosis	Death date prior to the first date of dementia diagnosis; transferred out date prior to the first date of dementia diagnosis

Supplementary Table 6. Baseline characteristics by coronary heart disease or peripheral artery disease

	Coronary heart disease, number (%)				Peripheral artery disease, number (%)			
	Presence		Absence		Presence		Absence	
Number of patients	14 880		49 079		3886		60 073	
Age, median (IQR)	78	(70-84)	73	(63-82)	77	(70-84)	74	(64-83)
Female	6468	(43.5)	24 989	(50.9)	1541	(39.7)	29 916	(49.8)
IMD Group 1 (least deprived)	2960	(19.9)	10 438	(21.3)	681	(17.5)	12 717	(21.2)
Group 2	2829	(19.0)	9122	(18.6)	710	(18.3)	11 241	(18.7)
Group 3	3103	(20.8)	10 675	(21.7)	825	(21.2)	12 953	(21.6)
Group 4	3049	(20.5)	9974	(20.3)	797	(20.5)	12 226	(20.3)
Group 5	2939	(19.8)	8870	(18.1)	873	(22.5)	10 936	(18.2)
Smoking Current [a]	2260	(15.2)	10 365	(21.1)	1165	(30.0)	11 460	(19.1)
Former	6213	(41.7)	14 562	(29.7)	1649	(42.4)	19 126	(31.8)
Never	6365	(42.8)	23 797	(48.5)	1063	(27.4)	29 099	(48.4)
BMI, median (IQR) [b]	27.0	(24.0-30.5)	26.5	(23.4-30.0)	26.3	(23.2-29.8)	26.6	(23.6-30.1)
Ischemic stroke [c]	13 740	(92.3)	44 637	(90.9)	3672	(94.5)	54 705	(91.6)
Intracerebral hemorrhage	1140	(7.7)	4442	(9.1)	214	(5.5)	5368	(8.9)
Atrial fibrillation	4842	(32.5)	8057	(16.4)	1069	(27.5)	11 830	(19.7)
Alcohol problems	673	(4.5)	2366	(4.8)	293	(7.5)	2746	(4.6)
Anxiety	3007	(20.2)	9134	(18.6)	762	(19.6)	11 379	(18.9)
Asthma	2388	(16.0)	5865	(12.0)	589	(15.2)	7664	(12.8)
COPD	2211	(14.9)	3999	(8.1)	781	(20.1)	5429	(9.0)
Coronary heart disease	14 880	(100.0)	0	(0)	1866	(48.0)	13 014	(21.7)
Depression	4380	(29.4)	12 594	(25.7)	1161	(29.9)	15 813	(26.3)
Diabetes	4212	(28.3)	7217	(14.7)	1287	(33.1)	10 142	(16.9)
Epilepsy	453	(3.0)	1567	(3.2)	119	(3.1)	1901	(3.2)
Hearing loss	4029	(27.1)	9965	(20.3)	1038	(26.7)	12 956	(21.6)
Heart failure	3346	(22.5)	2126	(4.3)	699	(18.0)	4773	(7.9)
Hyperlipidemia	7062	(47.5)	10 581	(21.6)	1740	(44.8)	15 903	(26.5)
Hypertension	10 907	(73.3)	26 402	(53.8)	2905	(74.8)	34 404	(57.3)
Parkinson’s disease	250	(1.7)	578	(1.2)	56	(1.4)	772	(1.3)
Peripheral artery disease	1866	(12.5)	2020	(4.1)	3886	(100.0)	0	(0)
Rheumatoid arthritis	1229	(8.3)	2956	(6.0)	332	(8.5)	3853	(6.4)
Transient ischemic attack	1847	(12.4)	5115	(10.4)	496	(12.8)	6466	(10.8)
Consultation, median (IQR)	44	(30-62)	30	(17-46)	45	(31-64)	32	(19-49)
Statins	10 677	(71.8)	14 724	(30.0)	2659	(68.4)	22 742	(37.9)
Other lipid-lowering drugs	1036	(7.0)	832	(1.7)	225	(5.8)	1643	(2.7)
Anticoagulant	1993	(13.4)	2924	(6.0)	473	(12.2)	4444	(7.4)
Antidiabetic drugs	3211	(21.6)	5335	(10.9)	1016	(26.1)	7530	(12.5)
Antihypertensive drugs	13 227	(88.9)	27 699	(56.4)	3191	(82.1)	37 735	(62.8)
Antiplatelet	10 992	(73.9)	14 443	(29.4)	2731	(70.3)	22 704	(37.8)

[a] A total of 397 (0.62%) patients had missing value of smoking status: 42 (0.28%), 355 (0.72%), 9 (0.23%), and 388 (0.65%) for CHD, no CHD, PAD, and no PAD, respectively.

[b] A total of 5,924 (9.26%) patients had missing value of BMI: 656 (4.41%), 5,221 (10.64%), 222 (5.71%), and 5,655 (9.41%) for CHD, no CHD, PAD, and no PAD, respectively.

[c] A total of 28,000 (43.78%) patients had an unspecified stroke subtype: 6,179 (41.53%), 21,821 (44.46%), 1,657 (42.64%), and 26,343 (43.85%) in CHD, no CHD, PAD, and no PAD group, respectively.

BMI, body mass index; CHD, coronary heart disease; COPD, chronic obstructive pulmonary disease; IMD, Index of Multiple Deprivation; IQR, interquartile range; PAD, peripheral artery disease.

Supplementary Table 7. Baseline characteristics of patients with complete and incomplete baseline data

	Baseline information, number (%)			
	Complete		Incomplete	
Number of patients	57 902		6057	
Age, median (IQR)	75	(65-82)	75	(62-85)
Female	28 488	(49.2)	2969	(49.0)
IMD Group 1 (least deprived)	12 073	(20.9)	1325	(21.9)
Group 2	10 737	(18.5)	1214	(20.0)
Group 3	12 392	(21.4)	1386	(22.9)
Group 4	11 803	(20.4)	1220	(20.1)
Group 5	10 897	(18.8)	912	(15.1)
Smoking Current [a]	11 206	(19.4)	1419	(23.4)
Former	19 506	(33.7)	1269	(21.0)
Never	27 190	(47.0)	2972	(49.1)
BMI, median (IQR) [b]	26.6	(23.6-30.1)	26.7	(24.1-30.9)
Ischemic stroke [c]	52 974	(91.5)	5403	(89.2)
Intracerebral hemorrhage	4928	(8.5)	654	(10.8)
Atrial fibrillation	11 863	(20.5)	1036	(17.1)
Alcohol problems	2739	(4.7)	300	(5.0)
Anxiety	11 349	(19.6)	792	(13.1)
Asthma	7883	(13.6)	370	(6.1)
COPD	5940	(10.3)	270	(4.5)
Coronary heart disease	14 177	(24.5)	703	(11.6)
Depression	15 802	(27.3)	1172	(19.3)
Diabetes	11 138	(19.2)	291	(4.8)
Epilepsy	1831	(3.2)	189	(3.1)
Hearing loss	13 023	(22.5)	971	(16.0)
Heart failure	5047	(8.7)	425	(7.0)
Hyperlipidemia	17 003	(29.4)	640	(10.6)
Hypertension	34 876	(60.2)	2433	(40.2)
Parkinson's disease	728	(1.3)	100	(1.7)
Peripheral artery disease	3651	(6.3)	235	(3.9)
Rheumatoid arthritis	3899	(6.7)	286	(4.7)
Transient ischemic attack	6435	(11.1)	527	(8.7)
Consultation, median (IQR)	34	(21-51)	22	(8-39)
Statins	24 271	(41.9)	1130	(18.7)
Other lipid-lowering drugs	1826	(3.2)	42	(0.7)
Anticoagulant	4645	(8.0)	272	(4.5)
Antidiabetic drugs	8403	(14.5)	143	(2.4)
Antihypertensive drugs	38 310	(66.2)	2616	(43.2)
Antiplatelet	23 771	(41.1)	1664	(27.5)

[a] A total of 5,660 (93.4%) patients with incomplete BMI values provided information on smoking status.
[b] A total of 133 (2.2%) patients with incomplete smoking information provided BMI data.
[c] A total of 28,000 (43.78%) patients had an unspecified stroke subtype: 25,477 (44.00%) and 2,523 (42.65%) patients with complete and incomplete baseline data, respectively.
BMI, body mass index; COPD, chronic obstructive pulmonary disease; IMD, Index of Multiple Deprivation; IQR, interquartile range.

Supplementary Table 8. Baseline characteristics stratified according to follow-up status

Characteristics, number (%) or median (IQR)	Survival		Death		Transfer-out from CPRD	
Number of patients	39 140		17 373		7446	
Age, median (IQR)	72	(62-80)	80	(73-87)	75	(62-84)
Female	18 564	(47.4)	8955	(51.5)	3938	(52.9)
IMD Group 1 (least deprived)	8208	(21.0)	3558	(20.5)	1632	(21.9)
Group 2	7419	(19.0)	3205	(18.4)	1327	(17.8)
Group 3	8294	(21.2)	3828	(22.0)	1656	(22.2)
Group 4	7938	(20.3)	3610	(20.8)	1475	(19.8)
Group 5	7281	(18.6)	3172	(18.3)	1356	(18.2)
Smoking Current [a]	7905	(20.2)	3067	(17.7)	1653	(22.2)
Former	11 823	(30.2)	6622	(38.1)	2330	(31.3)
Never	19 250	(49.2)	7507	(43.2)	3405	(45.7)
BMI, median (IQR) [b]	26.9	(24.0-30.1)	25.8	(22.7-29.4)	26.6	(23.4-30.0)
Ischemic stroke [c]	36 232	(92.5)	15 396	(88.6)	6749	(90.6)
Intracerebral hemorrhage	2908	(7.4)	1977	(11.4)	697	(9.4)
Atrial fibrillation	6489	(16.6)	4972	(28.6)	1438	(19.3)
Alcohol problems	1878	(4.8)	786	(4.5)	375	(5.0)
Anxiety	7848	(20.1)	2933	(16.9)	1360	(18.3)
Asthma	5143	(13.1)	2236	(12.9)	874	(11.7)
COPD	3181	(8.1)	2455	(14.1)	574	(7.7)
Coronary heart disease	7960	(20.3)	5349	(30.8)	1571	(21.1)
Depression	10 698	(27.3)	4201	(24.2)	2075	(27.9)
Diabetes	6461	(16.5)	3660	(21.1)	1308	(17.6)
Epilepsy	1199	(3.1)	551	(3.2)	270	(3.6)
Hearing loss	8046	(20.6)	4448	(25.6)	1500	(20.1)
Heart failure	2250	(5.7)	2688	(15.5)	534	(7.2)
Hyperlipidemia	11 288	(28.8)	4509	(26.0)	1846	(24.8)
Hypertension	21 982	(56.2)	11 107	(63.9)	4220	(56.7)
Parkinson's disease	367	(0.9)	350	(2.0)	111	(1.5)
Peripheral artery disease	1878	(4.8)	1590	(9.2)	418	(5.6)
Rheumatoid arthritis	2300	(5.9)	1414	(8.1)	471	(6.3)
Transient ischemic attack	4556	(11.6)	1663	(9.6)	743	(10.0)
Consultation, median (IQR)	31	(18-46)	40	(26-59)	31	(18-48)
Pre-stroke medication						
Statins	15 745	(40.2)	6982	(40.2)	2674	(35.9)
Other lipid-lowering drugs	1145	(2.9)	516	(3.0)	207	(2.8)
Anticoagulant	2485	(6.3)	1928	(11.1)	504	(6.8)
Antidiabetic drugs	4852	(12.4)	2708	(15.6)	986	(13.2)
Antihypertensive drugs	23 487	(60.0)	12 848	(74.0)	4591	(61.7)
Antiplatelet	14 573	(37.2)	8055	(46.4)	2807	(37.7)

[a] A total of 397 (0.62%) patients had missing value of smoking status: 162 (0.41%), 177 (1.02%), and 58 (0.78%) for survival, death and transfer-out, respectively.

[b] A total of 5924 (9.26%) patients had missing value of BMI: 3,222 (8.23%), 1,944 (11.19%), and 758 (10.18%) for survival, death and transfer-out, respectively.

[c] A total of 28,000 (43.78%) patients had an unspecified stroke subtype: 16,986 (43.40%), 7,865 (45.27%), and 3,149 (42.29%) for survival, death and transfer-out, respectively.

BMI, body mass index; COPD, chronic obstructive pulmonary disease; CPRD, Clinical Practice Research Datalink; IQR, interquartile range.

Supplementary Table 9. Association of prior atherosclerotic cardiovascular disease with dementia after stroke: stratified by age of stroke

	**Prior ASCVD**		**Prior CHD**		**Prior PAD**	
	Presence	Absence	Presence	Absence	Presence	Absence
**18-64 years**						
Total	2310	13 686	1981	14 015	552	15 444
Cases with dementia						
Cases with dementia	67	230	51	246	26	271
Person-years	11 821	74 776	10 268	76 329	2591	84 006
Rate [a]	5.7	3.1	5.0	3.2	10.0	3.2
Crude HR*	1.86 (1.41-2.43)	Reference	1.52 (1.13-2.05)	Reference	3.16 (2.09-4.78)	Reference
Adjusted HR*						
Model 1 [b]	1.53 (1.15-2.02)	Reference	1.29 (0.95-1.75)	Reference	2.28 (1.47-3.55)	Reference
Model 2 [c]	1.26 (0.92-1.72)	Reference	0.96 (0.67-1.36)	Reference	2.03 (1.29-3.18)	Reference
Model 3 [d]	1.16 (0.84-1.60)	Reference	0.88 (0.61-1.27)	Reference	1.92 (1.22-3.02)	Reference
**65-74 years**						
Total	3983	11 710	3457	12 236	991	14 702
Cases with dementia	381	953	329	1005	98	1236
Person-years	17 724	58 332	15 558	60 499	4021	72 036
Rate [a]	21.5	16.3	21.1	16.6	24.4	17.2
Crude HR*	1.29 (1.14-1.47)	Reference	1.25 (1.10-1.43)	Reference	1.40 (1.12-1.75)	Reference
Adjusted HR*						
Model 1 [b]	1.26 (1.10-1.43)	Reference	1.22 (1.07-1.39)	Reference	1.34 (1.06-1.67)	Reference
Model 2 [c]	1.14 (1.00-1.31)	Reference	1.10 (0.95-1.26)	Reference	1.17 (0.93-1.47)	Reference
Model 3 [d]	1.06 (0.91-1.23)	Reference	1.02 (0.87-1.19)	Reference	1.12 (0.89-1.40)	Reference
**75-84 years**						
Total	6438	13 454	5723	14 169	1475	18 417
Cases with dementia	1110	2258	987	2381	245	3123
Person-years	22 242	53 142	19 829	55 555	4669	70 716
Rate [a]	50.0	42.5	49.8	42.9	52.5	44.2
Crude HR*	1.16 (1.07-1.25)	Reference	1.15 (1.06-1.24)	Reference	1.17 (1.02-1.34)	Reference
Adjusted HR*						
Model 1 [b]	1.16 (1.07-1.25)	Reference	1.15 (1.06-1.25)	Reference	1.17 (1.02-1.35)	Reference
Model 2 [c]	1.06 (0.98-1.15)	Reference	1.05 (0.96-1.14)	Reference	1.07 (0.93-1.23)	Reference
Model 3 [d]	1.05 (0.96-1.15)	Reference	1.04 (0.95-1.15)	Reference	1.04 (0.90-1.20)	Reference
**≥85 years**						
Total	4169	8209	3719	8659	868	11 510
Cases with dementia	742	1524	671	1595	150	2116
Person-years	8456	19 888	7602	20 732	1595	26 738
Rate [a]	87.7	76.7	88.3	76.9	94.0	79.1
Crude HR*	1.11 (1.02-1.22)	Reference	1.10 (1.00-1.22)	Reference	1.22 (1.03-1.43)	Reference
Adjusted HR*						
Model 1 [b]	1.13 (1.03-1.24)	Reference	1.12 (1.02-1.23)	Reference	1.22 (1.04-1.44)	Reference
Model 2 [c]	1.07 (0.96-1.18)	Reference	1.05 (0.94-1.16)	Reference	1.17 (0.99-1.38)	Reference
Model 3 [d]	1.08 (0.97-1.20)	Reference	1.06 (0.95-1.19)	Reference	1.15 (0.97-1.36)	Reference

*Of 63,959 patients in total, 57,902 patients (14,182, 14,540, 18,407, and 10,773 for each age group, respectively) with complete baseline data were included in all the models (6057 were excluded due to missing value in smoking or BMI).

[a] Event rates reported in 1000 person-years.

[b] Model 1: adjusted for age (cubic spline variables), gender, IMD, smoking, and BMI (cubic spline variables).

[c] Model 2: adjusted for the variables in model 1 plus comorbidities (stroke subtype, atrial fibrillation, alcohol problem, anxiety, rheumatoid arthritis, asthma, chronic obstructive pulmonary disease, depression, diabetes, epilepsy, hearing loss, heart failure, hyperlipidemia, hypertension, Parkinson's disease, and transient ischemic attack. The CHD and PAD models additionally adjusted for PAD and CHD, respectively.
[d] Model 3: adjusted for the variables in model 2 plus consultation (cubic spline variables) and medications (statins, other lipid-lowering drugs, anticoagulant, antiplatelet, antihypertensive drugs, and antidiabetic drugs).
BMI, body mass index; CHD, coronary heart disease; IMD, Index of Multiple Deprivation; PAD, peripheral artery disease.

Supplementary Table 10. Association of prior atherosclerotic cardiovascular disease with dementia after stroke: stratified by gender

	Prior ASCVD		Prior CHD		Prior PAD	
	Presence	Absence	Presence	Absence	Presence	Absence
**Female**						
Total	7357	24 100	6468	24 989	1541	29 916
Cases with dementia	1138	2932	1004	3066	228	3842
Person-years	24 310	100 392	21 534	103 168	4596	120 107
Rate [a]	46.8	29.2	46.6	29.7	49.6	32.0
Crude HR*	1.56 (1.45-1.68)	Reference	1.52 (1.41-1.64)	Reference	1.51 (1.31-1.73)	Reference
Adjusted HR*						
Model 1 [b]	1.19 (1.10-1.27)	Reference	1.16 (1.08-1.25)	Reference	1.22 (1.06-1.40)	Reference
Model 2 [c]	1.08 (1.00-1.17)	Reference	1.06 (0.98-1.14)	Reference	1.11 (0.97-1.29)	Reference
Model 3 [d]	1.07 (0.98-1.16)	Reference	1.04 (0.96-1.13)	Reference	1.08 (0.94-1.25)	Reference
**Male**						
Total	9543	22 959	8412	24 090	2345	30 157
Cases with dementia	1162	2033	1034	2161	291	2904
Person-years	35 934	105 735	31 723	109 946	8280	133 389
Rate [a]	32.3	19.2	32.6	19.7	35.1	21.8
Crude HR*	1.60 (1.48-1.72)	Reference	1.57 (1.45-1.69)	Reference	1.56 (1.37-1.77)	Reference
Adjusted HR*						
Model 1 [b]	1.18 (1.09-1.27)	Reference	1.16 (1.07-1.25)	Reference	1.27 (1.12-1.45)	Reference
Model 2 [c]	1.09 (1.00-1.18)	Reference	1.06 (0.97-1.15)	Reference	1.15 (1.01-1.32)	Reference
Model 3 [d]	1.06 (0.97-1.16)	Reference	1.04 (0.95-1.14)	Reference	1.12 (0.98-1.28)	Reference

*Of 63,959 patients in total, 57,902 patients (28,488 for female and 29,414 for male, respectively) with complete baseline data were included in all the models (6,057 were excluded due to missing value in smoking or BMI).

[a] Event rates reported in 1000 person-years.

[b] Model 1: adjusted for age (cubic spline variables), gender, IMD, smoking, and BMI (cubic spline variables).

[c] Model 2: adjusted for the variables in model 1 plus comorbidities (stroke subtype, atrial fibrillation, alcohol problem, anxiety, rheumatoid arthritis, asthma, chronic obstructive pulmonary disease, depression, diabetes, epilepsy, hearing loss, heart failure, hyperlipidemia, hypertension, Parkinson's disease, and transient ischemic attack. The CHD and PAD models additionally adjusted for PAD and CHD, respectively.

[d] Model 3: adjusted for the variables in model 2 plus consultation (cubic spline variables) and medications (statins, other lipid-lowering drugs, anticoagulant, antiplatelet, antihypertensive drugs, and antidiabetic drugs).

BMI, body mass index; CHD, coronary heart disease; IMD, Index of Multiple Deprivation; PAD, peripheral artery disease.

Supplementary Table 11. Crude and partially adjusted association between the age of prior atherosclerotic cardiovascular disease onset and dementia after stroke

	**Crude estimate** [a]		**Adjusted estimate (Model 1)** [b]		**Adjusted estimate (Model 2)** [c]	
	Hazard ratio (95% CI)*	p-value for trend**	Hazard ratio (95% CI)*	p-value for trend**	Hazard ratio (95% CI)*	p-value for trend**
**Prior ASCVD**		<0.001		0.22		0.45
Absence	Reference		Reference		Reference	
<45	0.46 (0.33-0.63)		1.22 (0.88-1.68)		1.08 (0.78-1.49)	
45-65	1.06 (0.99-1.15)		1.26 (1.16-1.36)		1.15 (1.05-1.25)	
≥66	2.18 (2.06-2.32)		1.14 (1.07-1.21)		1.06 (0.99-1.13)	
**Prior CHD**		<0.001		0.12		0.25
Absence	Reference		Reference		Reference	
<45	0.47 (0.34-0.64)		1.22 (0.88-1.70)		1.07 (0.77-1.48)	
45-65	1.07 (0.99-1.16)		1.24 (1.14-1.35)		1.12 (1.02-1.22)	
≥66	2.14 (2.02-2.28)		1.12 (1.05-1.19)		1.03 (0.96-1.10)	
**Prior PAD**		0.02		0.98		0.93
Absence	Reference		Reference		Reference	
<45	0.13 (0.02-0.93)		0.64 (0.09-4.81)		0.57 (0.08-4.26)	
45-65	0.83 (0.69-1.00)		1.43 (1.18-1.73)		1.27 (1.05-1.55)	
≥66	1.99 (1.80-2.21)		1.20 (1.08-1.34)		1.11 (0.99-1.23)	

* Of 63,959 patients in total, 57,902 patients with complete baseline data were included in all the models (6057 were excluded due to missing value in smoking or BMI).

** Tests for linear trend were conducted in patients with ASCVD/CHD/PAD only, by assigning the medians (ASCVD: 41, 58, and 74; CHD: 41, 58, and 74; PAD: 42, 59, and 75) to the age levels of onset from the lowest to the highest and treating the variable as a numerical variable in the Cox models.

[a] No confounding variables were adjusted for.

[b] Model 1: adjusted for age (cubic spline variables), gender, IMD, smoking, and BMI (cubic spline variables).

[c] Model 2: adjusted for the variables in model 1 plus comorbidities (stroke subtype, atrial fibrillation, alcohol problem, anxiety, rheumatoid arthritis, asthma, chronic obstructive pulmonary disease, depression, diabetes, epilepsy, hearing loss, heart failure, hyperlipidemia, hypertension, Parkinson's disease, and transient ischemic attack. The CHD and PAD models additionally adjusted for PAD and CHD, respectively.

ASCVD, atherosclerotic cardiovascular disease; BMI, body mass index; CHD, coronary heart disease; HR: hazard ratio; IMD, Index of Multiple Deprivation; PAD, peripheral artery disease.

Supplementary Table 12. Crude and partially adjusted association between length of time with prior atherosclerotic cardiovascular disease and dementia after stroke

	Crude estimate [a]		Adjusted estimate (Model 1) [b]		Adjusted estimate (Model 2) [c]	
	Hazard ratio (95% CI)*	p-value for trend**	Hazard ratio (95% CI)*	p-value for trend**	Hazard ratio (95% CI)*	p-value for trend**
**Prior ASCVD**		<0.001		0.10		0.31
Absence	Reference		Reference		Reference	
Tertile 1 (1-7)	1.30 (1.20-1.41)		1.15 (1.06-1.25)		1.07 (0.99-1.17)	
Tertile 2 (8-14)	1.52 (1.40-1.64)		1.15 (1.06-1.25)		1.06 (0.97-1.15)	
Tertile 3 (≥15)	1.83 (1.69-1.98)		1.24 (1.15-1.33)		1.12 (1.04-1.22)	
**Prior CHD**		<0.001		0.04		0.14
Absence	Reference		Reference		Reference	
Tertile 1 (1-7)	1.25 (1.14-1.36)		1.11 (1.02-1.22)		1.03 (0.94-1.13)	
Tertile 2 (8-15)	1.48 (1.37-1.61)		1.14 (1.05-1.23)		1.03 (0.95-1.12)	
Tertile 3 (>=16)	1.85 (1.70-2.01)		1.23 (1.14-1.34)		1.11 (1.02-1.21)	
**Prior PAD**		0.06		0.90		0.90
Absence	Reference		Reference		Reference	
Tertile 1 (1-5)	1.39 (1.19-1.61)		1.27 (1.10-1.48)		1.18 (1.02-1.38)	
Tertile 2 (6-10)	1.39 (1.18-1.65)		1.20 (1.01-1.43)		1.08 (0.91-1.29)	
Tertile 3 (≥11)	1.70 (1.44-2.01)		1.26 (1.06-1.48)		1.14 (0.96-1.35)	

* Of 63,959 patients in total, 57,902 patients with complete baseline data were included in all the models (6,057 were excluded due to missing value in smoking or BMI).

** Tests for linear trend were conducted in patients with ASCVD/CHD/PAD only, by assigning the medians (ASCVD: 3, 11, and 20; CHD: 3, 11, and 21; PAD: 3, 8, and 15) to the length tertile levels from the lowest to the highest and treating the variable as a numerical variable in the Cox models.

[a] No confounding variables were adjusted for.

[b] Model 1: adjusted for age (cubic spline variables), gender, IMD, smoking, and BMI (cubic spline variables).

[c] Model 2: adjusted for the variables in model 1 plus comorbidities (stroke subtype, atrial fibrillation, alcohol problem, anxiety, rheumatoid arthritis, asthma, chronic obstructive pulmonary disease, depression, diabetes, epilepsy, hearing loss, heart failure, hyperlipidemia, hypertension, Parkinson's disease, and transient ischemic attack. The CHD and PAD models additionally adjusted for PAD and CHD, respectively.

ASCVD, atherosclerotic cardiovascular disease; BMI, body mass index; CHD, coronary heart disease; HR: hazard ratio; IMD, Index of Multiple Deprivation; PAD, peripheral artery disease.

Supplementary Table 13. Sensitivity analysis excluding patients having a first record of dementia within the first 6-month follow-up

	Prior ASCVD, HR (95%CI)	Prior CHD, HR (95%CI)	Prior PAD, HR (95%CI)
**Crude estimate***	1.52 (1.43-1.61)	1.48 (1.39-1.57)	1.50 (1.34-1.67)
**Adjusted estimate***			
Model 1 [a]	1.18 (1.11-1.25)	1.15 (1.08-1.22)	1.28 (1.14-1.43)
Model 2 [b]	1.09 (1.03-1.16)	1.05 (0.98-1.12)	1.18 (1.05-1.32)
Model 3 [c]	1.07 (1.00-1.14)	1.03 (0.96-1.10)	1.14 (1.01-1.28)
**Age of onset***[c]			
<45 years	1.15 (0.80-1.63)	1.12 (0.78-1.61)	0.72 (0.10-5.43)
45-65	1.11 (1.01-1.23)	1.07 (0.96-1.19)	1.32 (1.07-1.64)
>65	1.04 (0.97-1.13)	1.00 (0.93-1.09)	1.09 (0.96-1.24)
p-value for trend**	0.47	0.33	0.55
**Length of history***[c]			
Tertile 1 (lowest)	1.04 (0.95-1.15)	0.98 (0.88-1.09)	1.19 (1.00-1.41)
Tertile 2	1.06 (0.96-1.17)	1.03 (0.93-1.14)	1.03 (0.85-1.26)
Tertile 3	1.11 (1.01-1.22)	1.08 (0.97-1.20)	1.18 (0.98-1.43)
p-value for trend***	0.20	0.09	0.85

*1,518 patients with occurrence of dementia during the first 6 months of stroke were excluded. Of the remaining 62,441 patients in total, 56,533 patients with complete baseline data were included in all the models (5,908 were excluded due to missing value in smoking or BMI). For the adjusted estimates, 15,553 and 40,980 patients were in the ASCVD and no ASCVD group; 13,732 and 42,801 patients were in the CHD and no CHD group; 3,538 and 52,995 patients in the PAD and no PAD group, respectively.

**Tests for linear trend were conducted in patients with ASCVD/CHD/PAD only, by assigning the medians (ASCVD: 41, 58, and 74; CHD: 41, 58, and 74; PAD: 42, 59, and 75) to the age levels of onset from the lowest to the highest and treating the variable as a numerical v ariable in the Cox models.

***Tests for linear trend were conducted in patients with ASCVD/CHD/PAD only, by assigning the medians (ASCVD: 3, 11, and 20; CHD: 3, 11, and 21; PAD: 3, 8, and 15) to the length tertile levels from the lowest to the highest and treating the variable as a continuous variable in the Cox models.

[a] Model 1: adjusted for age (cubic spline variables), gender, IMD, smoking, and BMI (cubic spline variables).

[b] Model 2: adjusted for the variables in model 1 plus comorbidities (stroke subtype, atrial fibrillation, alcohol problem, anxiety, rheumatoid arthritis, asthma, chronic obstructive pulmonary disease, depression, diabetes, epilepsy, hearing loss, heart failure, hyperlipidemia, hypertension, Parkinson's disease, and transient ischemic attack. The CHD and PAD models additionally adjusted for PAD and CHD, respectively.

[c] Model 3: adjusted for the variables in model 2 plus consultation (cubic spline variables) and medications (statins, other lipid-lowering drugs, anticoagulant, antiplatelet, antihypertensive drugs, and antidiabetic drugs).

ASCVD, atherosclerotic cardiovascular disease; BMI, body mass index; CHD, coronary heart disease; HR, hazard ratio; IMD, Index of Multiple Deprivation; PAD, peripheral artery disease

Supplementary Table 14. Sensitivity analysis separating unspecified stroke from ischemic stroke

	Prior ASCVD, HR (95%CI)	Prior CHD, HR (95%CI)	Prior PAD, HR (95%CI)
**Crude estimate***	1.52 (1.45-1.61)	1.50 (1.42-1.58)	1.47 (1.34-1.62)
**Adjusted estimate***			
Model 1 [a]	1.18 (1.12-1.25)	1.16 (1.10-1.23)	1.25 (1.13-1.37)
Model 2 [b]	1.08 (1.02-1.15)	1.05 (0.99-1.12)	1.14 (1.03-1.26)
Model 3 [c]	1.06 (1.00-1.13)	1.04 (0.97-1.10)	1.10 (1.00-1.22)
**Age of onset***[c]			
<45 years	1.07 (0.77-1.49)	1.07 (0.77-1.49)	0.55 (0.07-4.16)
45-65	1.13 (1.03-1.23)	1.10 (1.01-1.21)	1.26 (1.03-1.53)
>65	1.03 (0.96-1.10)	1.00 (0.93-1.08)	1.06 (0.95-1.19)
p-value for trend**	0.32	0.18	0.99
**Length of history***[c]			
Tertile 1 (lowest)	1.03 (0.95-1.12)	1.00 (0.91-1.09)	1.12 (0.96-1.31)
Tertile 2	1.04 (0.95-1.14)	1.02 (0.94-1.12)	1.05 (0.88-1.24)
Tertile 3	1.10 (1.02-1.20)	1.09 (1.00-1.19)	1.13 (0.95-1.33)
p-value for trend***	0.15	0.07	0.95

*Of 63,959 patients in total, 57,902 patients with complete baseline data were included in all the models (6,057 were excluded due to missing value in smoking or BMI). For the adjusted estimates, 16,046 and 41,856 patients were in the ASCVD and no ASCVD group; 14,177 and 43,725 patients were in the CHD and no CHD group; 3651 and 54,251 patients in the PAD and no PAD group, respectively.

**Tests for linear trend were conducted in patients with ASCVD/CHD/PAD only, by assigning the medians (ASCVD: 41, 58, and 74; CHD: 41, 58, and 74; PAD: 42, 59, and 75) to the age levels of onset from the lowest to the highest and treating the variable as a continuous variable in the Cox models.

***Tests for linear trend were conducted in patients with ASCVD/CHD/PAD only, by assigning the medians (ASCVD: 3, 11, and 20; CHD: 3, 11, and 21; PAD: 3, 8, and 15) to the length tertile levels from the lowest to the highest and treating the variable as a numerical variable in the Cox models.

[a] Model 1: adjusted for age (cubic spline variables), gender, IMD, smoking, and BMI (cubic spline variables).

[b] Model 2: adjusted for the variables in model 1 plus comorbidities (stroke subtype, atrial fibrillation, alcohol problem, anxiety, rheumatoid arthritis, asthma, chronic obstructive pulmonary disease, depression, diabetes, epilepsy, hearing loss, heart failure, hyperlipidemia, hypertension, Parkinson's disease, and transient ischemic attack. The CHD and PAD models additionally adjusted for PAD and CHD, respectively.

[c] Model 3: adjusted for the variables in model 2 plus consultation (cubic spline variables) and medications (statins, other lipid-lowering drugs, anticoagulant, antiplatelet, antihypertensive drugs, and antidiabetic drugs).

ASCVD, atherosclerotic cardiovascular disease; BMI, body mass index; CHD, coronary heart disease; HR, hazard ratio; IMD, Index of Multiple Deprivation; PAD, peripheral artery disease.

Supplementary Table 15. Sensitivity analysis restricting to patients with linkage to Hospital Episode Statistics

	Prior ASCVD, HR (95%CI)	Prior CHD, HR (95%CI)	Prior PAD, HR (95%CI)
**Crude estimate***	1.46 (1.37-1.55)	1.42 (1.34-1.52)	1.44 (1.29-1.61)
**Adjusted estimate***			
Model 1 [a]	1.12 (1.06-1.19)	1.10 (1.03-1.17)	1.20 (1.07-1.35)
Model 2 [b]	1.06 (0.99-1.13)	1.02 (0.95-1.10)	1.13 (1.00-1.27)
Model 3 [c]	1.02 (0.95-1.10)	0.99 (0.92-1.07)	1.08 (0.96-1.22)
**Age of onset***[c]			
<45 years	0.88 (0.57-1.38)	0.88 (0.56-1.38)	Not estimated
45-65	1.12 (1.01-1.25)	1.09 (0.97-1.21)	1.25 (0.99-1.59)
>65	0.99 (0.91-1.07)	0.96 (0.88-1.04)	1.05 (0.92-1.19)
p-value for trend**	0.55	0.36	0.98
**Length of history***[c]			
Tertile 1 (lowest)	0.98 (0.89-1.09)	0.96 (0.86-1.07)	1.05 (0.86-1.29)
Tertile 2	1.02 (0.92-1.13)	0.97 (0.88-1.08)	1.09 (0.91-1.31)
Tertile 3	1.08 (0.97-1.19)	1.05 (0.95-1.17)	1.11 (0.92-1.33)
p-value for trend***	0.18	0.13	0.60

*28,891 patients with no linkage to HES were excluded. Of the remaining 35,068 patients in total, 31,623 patients with complete baseline data were included in all the adjustment models (3,445 were excluded due to missing value in smoking or BMI). For the adjusted estimates, 9,534 and 22,089 patients were in the ASCVD and no ASCVD group; 8,490 and 23,133 patients were in the CHD and no CHD group; 2,156 and 29,467 patients in the PAD and no PAD group, respectively.

**Tests for linear trend were conducted in patients with ASCVD/CHD/PAD only, by assigning the medians (ASCVD: 41, 58, and 75; CHD: 41, 58, and 75; PAD: 43, 59, and 75) to the age levels of onset from the lowest to the highest and treating the variable as a numerical v ariable in the Cox models.

***Tests for linear trend were conducted in patients with ASCVD/CHD/PAD only, by assigning the medians (ASCVD: 3, 10, and 19; CHD: 3, 10, and 20; PAD: 2, 7, and 14) to the length tertile levels from the lowest to the highest and treating the variable as a continuous variable in the Cox models.

[a] Model 1: adjusted for age (cubic spline variables), gender, IMD, smoking, and BMI (cubic spline variables).

[b] Model 2: adjusted for the variables in model 1 plus comorbidities (stroke subtype, atrial fibrillation, alcohol problem, anxiety, rheumatoid arthritis, asthma, chronic obstructive pulmonary disease, depression, diabetes, epilepsy, hearing loss, heart failure, hyperlipidemia, hypertension, Parkinson's disease, and transient ischemic attack. The CHD and PAD models additionally adjusted for PAD and CHD, respectively.

[c] Model 3: adjusted for the variables in model 2 plus consultation (cubic spline variables) and medications (statins, other lipid-lowering drugs, anticoagulant, antiplatelet, antihypertensive drugs, and antidiabetic drugs).

ASCVD, atherosclerotic cardiovascular disease; BMI, body mass index; CHD, coronary heart disease; IMD, Index of Multiple Deprivation; HES, Hospital Episode Statistics; HR: hazard ratio; PAD, peripheral artery disease.

Supplementary Table 16. Sensitivity analysis changing missing BMI to 5th or 95th percentile and changing missing smoking to never or current smoking

	5th Percentile BMI			95th Percentile BMI		
	Prior ASCVD, HR (95%CI)	Prior CHD, HR (95%CI)	Prior PAD, HR (95%CI)	Prior ASCVD, HR (95%CI)	Prior CHD, HR (95%CI)	Prior PAD, HR (95%CI)
Changing missing smoking to never smoking						
**Crude estimate***	1.54 (1.47-1.62)	1.52 (1.44-1.60)	1.46 (1.33-1.60)	1.54 (1.47-1.62)	1.52 (1.44-1.60)	1.46 (1.33-1.60)
**Adjusted estimate***						
Model 1 [a]	1.20 (1.14-1.26)	1.18 (1.12-1.25)	1.23 (1.12-1.35)	1.18 (1.12-1.24)	1.16 (1.10-1.23)	1.22 (1.11-1.34)
Model 2 [b]	1.10 (1.04-1.16)	1.08 (1.02-1.14)	1.12 (1.02-1.23)	1.09 (1.03-1.15)	1.07 (1.01-1.13)	1.12 (1.02-1.23)
Model 3 [c]	1.08 (1.02-1.15)	1.07 (1.00-1.14)	1.09 (0.99-1.20)	1.08 (1.02-1.14)	1.06 (1.00-1.13)	1.09 (0.99-1.20)
**Age of onset***[c]						
<45 years	1.11 (0.81-1.53)	1.11 (0.80-1.54)	0.56 (0.07-4.20)	1.10 (0.80-1.51)	1.10 (0.79-1.52)	0.55 (0.07-4.16)
45-65	1.15 (1.06-1.25)	1.13 (1.04-1.23)	1.25 (1.03-1.52)	1.15 (1.06-1.25)	1.12 (1.03-1.23)	1.26 (1.04-1.53)
>65	1.05 (0.98-1.12)	1.04 (0.97-1.11)	1.05 (0.94-1.16)	1.05 (0.98-1.12)	1.03 (0.96-1.11)	1.05 (0.94-1.17)
P-value for trend**	0.30	0.20	0.91	0.36	0.24	0.93
**Length of history***[c]						
Tertile 1 (lowest)	1.06 (0.97-1.16)	1.03 (0.94-1.13)	1.12 (0.97-1.30)	1.06 (0.97-1.16)	1.03 (0.94-1.13)	1.12 (0.97-1.30)
Tertile 2	1.07 (0.98-1.16)	1.06 (0.97-1.15)	1.02 (0.86-1.21)	1.06 (0.98-1.15)	1.05 (0.96-1.15)	1.03 (0.87-1.21)
Tertile 3	1.12 (1.04-1.22)	1.12 (1.03-1.22)	1.11 (0.94-1.30)	1.12 (1.03-1.21)	1.11 (1.02-1.21)	1.12 (0.95-1.31)
p-value for trend***	0.17	0.10	0.97	0.23	0.14	0.99
Changing missing smoking to current smoking						
**Crude estimate***	1.54 (1.47-1.62)	1.52 (1.44-1.60)	1.46 (1.33-1.60)	1.54 (1.47-1.62)	1.52 (1.44-1.60)	1.46 (1.33-1.60)
**Adjusted estimate***						
Model 1 [a]	1.20 (1.14-1.26)	1.18 (1.12-1.25)	1.23 (1.12-1.35)	1.18 (1.12-1.24)	1.16 (1.10-1.23)	1.22 (1.11-1.34)
Model 2 [b]	1.10 (1.04-1.16)	1.08 (1.02-1.14)	1.12 (1.02-1.23)	1.09 (1.03-1.15)	1.07 (1.01-1.13)	1.12 (1.02-1.23)
Model 3 [c]	1.08 (1.02-1.15)	1.07 (1.00-1.14)	1.09 (0.99-1.20)	1.08 (1.02-1.14)	1.06 (1.00-1.13)	1.09 (0.99-1.20)
**Age of onset***[c]						
<45 years	1.11 (0.81-1.53)	1.11 (0.80-1.54)	0.56 (0.07-4.20)	1.09 (0.80-1.51)	1.10 (0.79-1.52)	0.55 (0.07-4.16)
45-65	1.13 (1.06-1.25)	1.13 (1.04-1.23)	1.25 (1.03-1.52)	1.15 (1.05-1.25)	1.13 (1.03-1.23)	1.26 (1.04-1.53)
>65	1.05 (0.98-1.12)	1.04 (0.97-1.11)	1.05 (0.94-1.16)	1.05 (0.98-1.12)	1.03 (0.96-1.11)	1.05 (0.94-1.17)
p-value for trend**	0.31	0.20	0.91	0.36	0.24	0.93
**Length of history***[c]						
Tertile 1 (lowest)	1.06 (0.97-1.16)	1.03 (0.94-1.13)	1.12 (0.97-1.30)	1.06 (0.97-1.16)	1.03 (0.94-1.13)	1.12 (0.97-1.30)
Tertile 2	1.07 (0.98-1.16)	1.06 (0.97-1.15)	1.02 (0.86-1.21)	1.06 (0.97-1.15)	1.05 (0.96-1.15)	1.02 (0.86-1.21)
Tertile 3	1.12 (1.04-1.22)	1.12 (1.03-1.22)	1.11 (0.94-1.30)	1.12 (1.03-1.21)	1.11 (1.02-1.21)	1.11 (0.95-1.31)
p-value for trend***	0.17	0.10	0.97	0.24	0.14	0.99

* All eligible patients (n=63,959) were included in all the models: 16,900 and 47,059 patients were in the ASCVD and no ASCVD group; 14,880 and 49,079 patients were in the CHD and no CHD group; 3,886 and 60,073 patients in the PAD and no PAD group, respectively.

**Tests for linear trend were conducted in patients with ASCVD/CHD/PAD only, by assigning the medians (ASCVD: 41, 58, and 74; CHD: 41, 58, and 74; PAD: 42, 59 and 75) to the age levels of onset from the lowest to the highest and treating the variable as a numerical v ariable in the Cox models.

***Tests for linear trend were conducted in patients with ASCVD/CHD/PAD only, by assigning the medians (ASCVD: 3, 10, and 20; CHD: 3, 11, and 21; PAD: 3, 8, and 15) to the length tertile levels from the lowest to the highest and treating the variable as a continuous variable in the Cox models.

[a] Model 1: adjusted for age (cubic spline variables), gender, IMD, smoking, and BMI (cubic spline variables).

[b] Model 2: adjusted for the variables in model 1 plus comorbidities (stroke subtype, atrial fibrillation, alcohol problem, anxiety, rheumatoid arthritis, asthma, chronic obstructive pulmonary disease, depression, diabetes, epilepsy, hearing loss, heart failure, hyperlipidemia, hypertension, Parkinson's disease, and transient ischemic attack. The CHD and PAD models additionally adjusted for PAD and CHD, respectively.

[c] Model 3: adjusted for the variables in model 2 plus consultation (cubic spline variables) and medications (statins, other lipid-lowering drugs, anticoagulant, antiplatelet, antihypertensive drugs, and antidiabetic drugs)
ASCVD, atherosclerotic cardiovascular disease; BMI, body mass index; CHD, coronary heart disease; IMD, Index of Multiple Deprivation; HR: hazard ratio; PAD, peripheral artery disease.

Supplementary Table 17. Sensitivity analysis using competing-risks regression models

	Prior ASCVD, HR (95%CI)	Prior CHD, HR (95%CI)	Prior PAD, HR (95%CI)
**Crude estimate***	1.31 (1.24-1.38)	1.30 (1.23-1.37)	1.22 (1.11-1.34)
**Adjusted estimate***			
Model 1 [a]	1.05 (0.99-1.10)	1.04 (0.99-1.10)	1.06 (0.96-1.17)
Model 2 [b]	1.02 (0.96-1.08)	1.02 (0.96-1.08)	1.01 (0.92-1.12)
Model 3 [c]	1.01 (0.95-1.07)	1.01 (0.95-1.07)	1.00 (0.90-1.10)
**Age of onset***[c]			
<45 years	1.05 (0.76-1.44)	1.07 (0.77-1.48)	0.49 (0.07-3.58)
45-65	1.07 (0.98-1.17)	1.07 (0.98-1.17)	1.13 (0.93-1.38)
>65	0.98 (0.91-1.05)	0.98 (0.91-1.05)	0.96 (0.86-1.08)
p-value for trend**	0.67	0.32	0.52
**Length of history***[c]			
Tertile 1 (lowest)	0.99 (0.91-1.07)	0.97 (0.88-1.06)	1.06 (0.91-1.23)
Tertile 2	1.01 (0.92-1.10)	1.01 (0.93-1.11)	0.94 (0.80-1.12)
Tertile 3	1.04 (0.95-1.13)	1.05 (0.96-1.14)	0.97 (0.82-1.15)
p-value for trend***	0.27	0.13	0.53

*Of 63,959 patients in total, 57,902 patients with complete baseline data were included in all the models (6,057 were excluded due to missing value in smoking or BMI). For the adjusted estimates, 16,046 and 41,856 patients were in the ASCVD and no ASCVD group; 14,177 and 43,725 patients were in the CHD and no CHD group; 3,651 and 54,251 patients in the PAD and no PAD group, respectively.

**Tests for linear trend were conducted in patients with ASCVD/CHD/PAD only, by assigning the medians (ASCVD: 41, 58, and 74; CHD: 41, 58, and 74; PAD: 42, 59, and 75) to the age levels of onset from the lowest to the highest and treating the variable as a numerical v ariable in the competing-risks regression models.

***Tests for linear trend were conducted in patients with ASCVD/CHD/PAD only, by assigning the medians (ASCVD: 3, 11, and 20; CHD: 3, 11, and 21; PAD: 3, 8, and 15) to the length tertile levels from the lowest to the highest and treating the variable as a continuous variable in the competing-risks regression models.

[a] Model 1: adjusted for age (cubic spline variables), gender, IMD, smoking, and BMI (cubic spline variables).

[b] Model 2: adjusted for the variables in model 1 plus comorbidities (stroke subtype, atrial fibrillation, alcohol problem, anxiety, rheumatoid arthritis, asthma, chronic obstructive pulmonary disease, depression, diabetes, epilepsy, hearing loss, heart failure, hyperlipidemia, hypertension, Parkinson's disease, and transient ischemic attack. The CHD and PAD models additionally adjusted for PAD and CHD, respectively.

[c] Model 3: adjusted for the variables in model 2 plus consultation (cubic spline variables) and medications (statins, other lipid-lowering drugs, anticoagulant, antiplatelet, antihypertensive drugs, and antidiabetic drugs).

ASCVD, atherosclerotic cardiovascular disease; BMI, body mass index; CHD, coronary heart disease; IMD, Index of Multiple Deprivation; HR: hazard ratio; PAD, peripheral artery disease.

Supplementary Table 18. A series of sensitivity analyses on the interactions between coronary heart disease and peripheral artery disease in the risk of dementia after stroke

	Crude estimate, HR (95% CI)	Adjusted estimate, HR (95% CI)		
		Model 1 [a]	Model 2 [b]	Model 3 [c]
Excluding dementia occurring within the first 6-month follow-up [A]				
CHD only	1.49 (1.40-1.59)	1.15 (1.07-1.22)	1.06 (0.99-1.14)	1.04 (0.96-1.12)
PAD only	1.61 (1.40-1.85)	1.32 (1.15-1.53)	1.26 (1.09-1.45)	1.21 (1.04-1.39)
CHD plus PAD	1.68 (1.43-1.97)	1.33 (1.13-1.56)	1.16 (0.98-1.37)	1.11 (0.94-1.31)
p-interaction*	0.001	0.22	0.19	0.27
p-interaction**	0.019	0.31	0.22	0.28
Separating unspecified stroke from ischemic stroke [B]				
CHD only	1.50 (1.41-1.59)	1.15 (1.09-1.22)	1.06 (1.00-1.13)	1.04 (0.97-1.11)
PAD only	1.53 (1.36-1.73)	1.25 (1.10-1.41)	1.18 (1.04-1.33)	1.13 (0.99-1.28)
CHD plus PAD	1.72 (1.50-1.98)	1.35 (1.18-1.55)	1.16 (1.01-1.34)	1.12 (0.97-1.29)
p-interaction*	0.002	0.51	0.46	0.61
p-interaction**	0.04	0.69	0.50	0.63
Restricting to patients with linkage to HES data [C]				
CHD only	1.43 (1.33-1.53)	1.09 (1.02-1.17)	1.03 (0.96-1.11)	1.00 (0.93-1.08)
PAD only	1.54 (1.34-1.77)	1.25 (1.08-1.44)	1.19 (1.03-1.38)	1.14 (0.98-1.33)
CHD plus PAD	1.62 (1.38-1.90)	1.22 (1.04-1.43)	1.10 (0.93-1.30)	1.04 (0.88-1.23)
p-interaction*	0.005	0.31	0.29	0.39
p-interaction**	0.04	0.36	0.30	0.38
Changing missing BMI and smoking status to 5th percentile and never smoking respectively [D]				
CHD only	1.52 (1.44-1.61)	1.17 (1.11-1.24)	1.08 (1.02-1.15)	1.07 (1.00-1.14)
PAD only	1.51 (1.34-1.69)	1.22 (1.09-1.37)	1.15 (1.02-1.29)	1.11 (0.98-1.25)
CHD plus PAD	1.73 (1.51-1.98)	1.36 (1.19-1.55)	1.18 (1.03-1.35)	1.14 (0.99-1.31)
p-interaction*	0.002	0.53	0.52	0.65
p-interaction**	0.04	0.73	0.58	0.69
Changing missing BMI and smoking status to 5th percentile and current smoking respectively [D]				
CHD only	1.52 (1.44-1.61)	1.17 (1.11-1.24)	1.08 (1.02-1.15)	1.07 (1.00-1.14)
PAD only	1.51 (1.34-1.69)	1.22 (1.09-1.37)	1.15 (1.02-1.29)	1.11 (0.98-1.25)
CHD plus PAD	1.73 (1.51-1.98)	1.36 (1.19-1.55)	1.18 (1.03-1.35)	1.14 (0.99-1.31)
p-interaction*	0.002	0.53	0.52	0.65
p-interaction**	0.04	0.73	0.58	0.69
Changing missing BMI and smoking status to 95th percentile and never smoking respectively [D]				
CHD only	1.52 (1.44-1.61)	1.16 (1.09-1.22)	1.07 (1.01-1.14)	1.07 (0.99-1.14)
PAD only	1.51 (1.34-1.69)	1.21 (1.08-1.36)	1.15 (1.02-1.29)	1.11 (0.99-1.25)
CHD plus PAD	1.73 (1.51-1.98)	1.34 (1.17-1.53)	1.17 (1.02-1.34)	1.14 (0.99-1.31)
p-interaction*	0.002	0.59	0.57	0.68
p-interaction**	0.04	0.77	0.62	0.72
Changing missing BMI and smoking status to 95th percentile and current smoking respectively [D]				
CHD only	1.52 (1.44-1.61)	1.16 (1.09-1.22)	1.07 (1.01-1.14)	1.07 (1.00-1.14)
PAD only	1.51 (1.34-1.69)	1.21 (1.08-1.36)	1.15 (1.02-1.29)	1.11 (0.99-1.25)
CHD plus PAD	1.73 (1.51-1.98)	1.34 (1.17-1.53)	1.17 (1.02-1.34)	1.14 (0.99-1.31)
p-interaction*	0.002	0.59	0.57	0.68
p-interaction**	0.04	0.77	0.62	0.72
Using competing-risks regression models [B]				

CHD only	1.31 (1.23-1.39)	1.04 (0.98-1.11)	1.02 (0.96-1.09)	1.01 (0.95-1.08)
PAD only	1.27 (1.13-1.44)	1.07 (0.94-1.21)	1.03 (0.90-1.16)	1.00 (0.88-1.14)
CHD plus PAD	1.34 (1.17-1.53)	1.08 (0.94-1.23)	1.02 (0.89-1.18)	1.00 (0.87-1.15)
p-interaction*	0.02	0.75	0.80	0.92
p-interaction**	0.05	0.77	0.80	0.92

[A] Including 56 533 patients with complete baseline data: 40,980 patients without any ASCVDs (reference group), 13,836 with only one ASCVD (12,015 with CHD and 1,821 with PAD), and 1,717 with two ASCVDs (CHD plus PAD).

[B] Including 57,902 patients with complete baseline data: 41,856 patients without any ASCVDs (reference group), 14,264 with only one ASCVD (12,395 with CHD and 1,869 with PAD), and 1,782 with two ASCVDs (CHD plus PAD).

[C] Including 31,623 patients with complete baseline data: 22,089 patients without any ASCVDs (reference group), 8,422 with only one ASCVD (7,378 with CHD and 1,044 with PAD), and 1,112 with two ASCVDs (CHD plus PAD).

[D] Including all 63,959 patients: 47,059 patients without any ASCVDs (reference group), 15,034 with only one ASCVD (13,014 with CHD and 2,020 with PAD), and 1,866 with two ASCVDs (CHD plus PAD).

[a] Model 1: adjusted for age (cubic spline variables), gender, IMD, smoking, and BMI (cubic spline variables).

[b] Model 2: adjusted for the variables in model 1 plus comorbidities (stroke subtype, atrial fibrillation, alcohol problem, anxiety, rheumatoid arthritis, asthma, chronic obstructive pulmonary disease, depression, diabetes, epilepsy, hearing loss, heart failure, hyperlipidemia, hypertension, Parkinson’s disease, and transient ischemic attack.

[c] Model 3: adjusted for the variables in model 2 plus consultation (cubic spline variables) and medications (statins, other lipid-lowering drugs, anticoagulant, antiplatelet, antihypertensive drugs, and antidiabetic drugs).

A product term for CHD and PAD was included in all the models.

*p-value for testing multiplicative interaction between CHD and PAD.

**p-value for testing additive interaction between CHD and PAD.

ASCVD, atherosclerotic cardiovascular disease; BMI, body mass index; CHD, coronary heart disease; HR, hazard ratio; IMD, Index of Multiple Deprivation; PAD, peripheral artery disease.

Supplementary Figure 1. Flow chart of patient inclusion

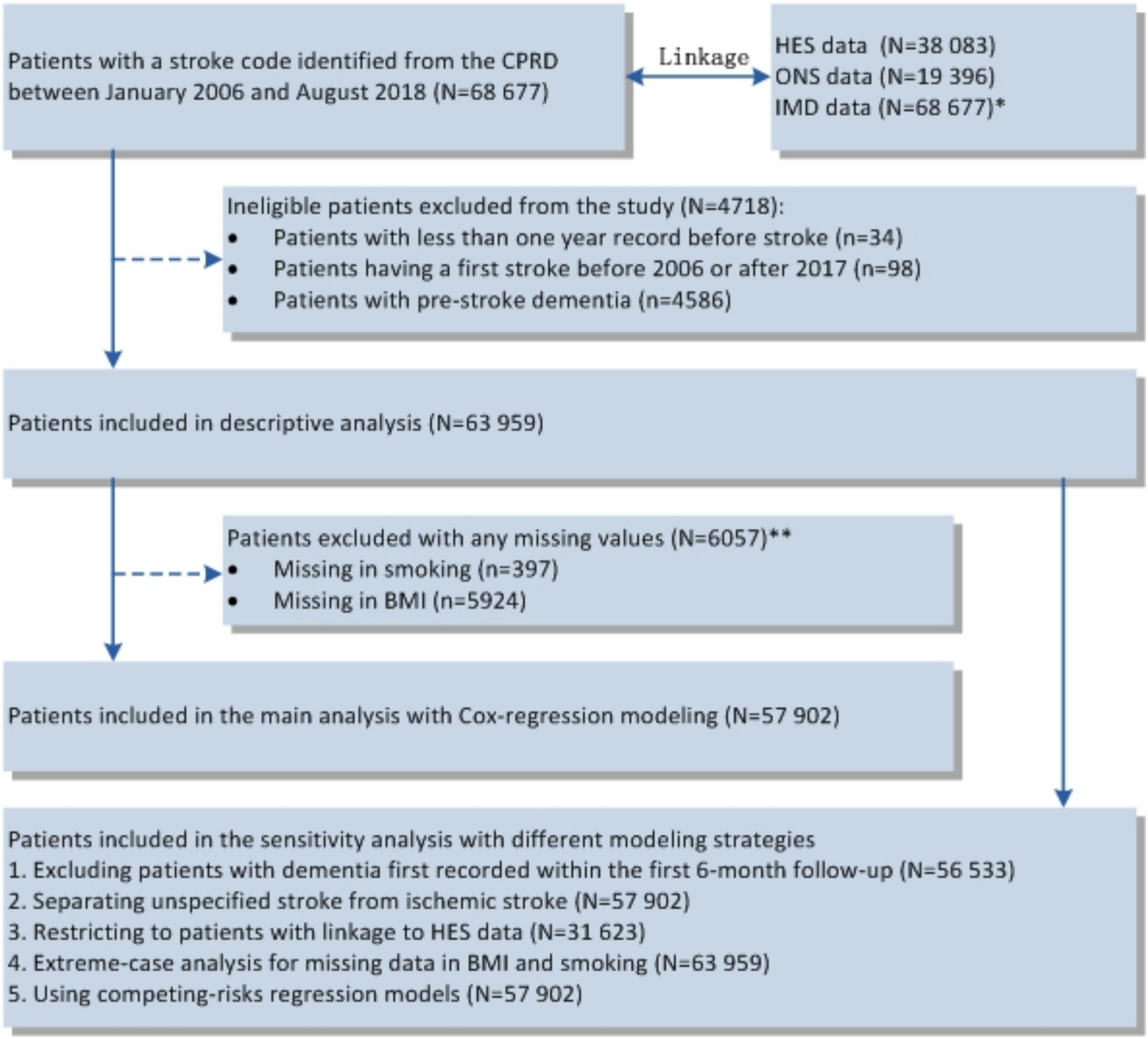

*In the IMD dataset, 38,616 patients had patient-level IMD and the other 30,061 patients had practice-level IMD.

**Missingness in smoking and BMI was not mutually exclusive.

BMI, body mass index; CPRD, Clinical Practice Research Datalink; HES, Hospital Episode Statistics; IMD, Index of Multiple Deprivation; ONS, Office for National Statistics.

Supplementary Figure 2. Kaplan-Meier plots

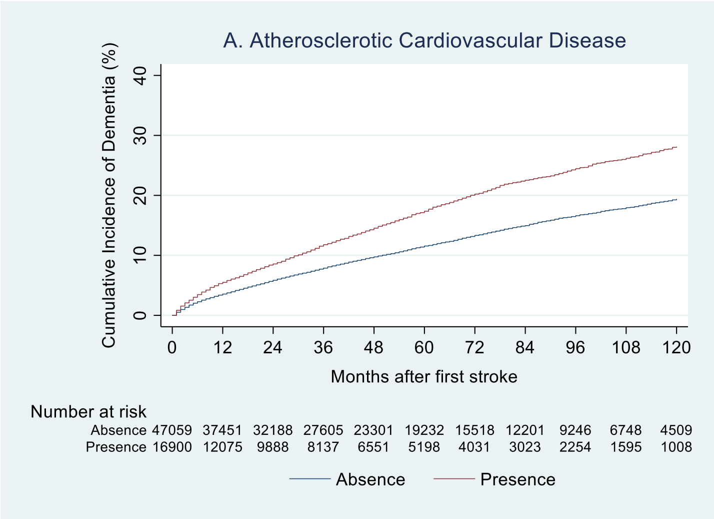

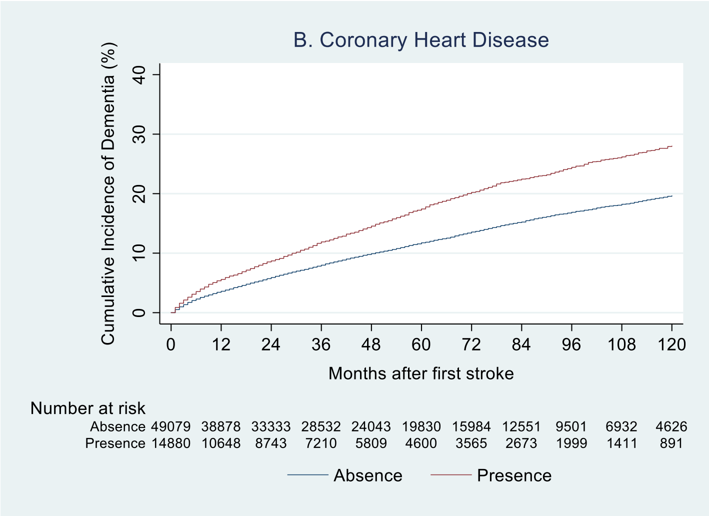

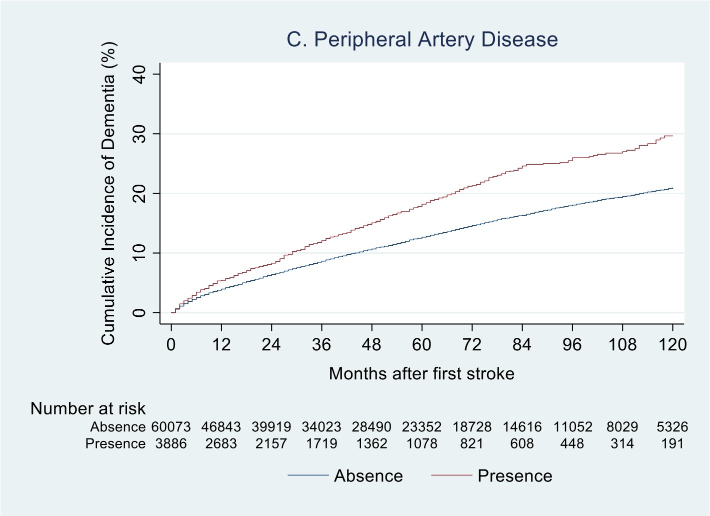

Supplementary Figure 3. Log-log plots

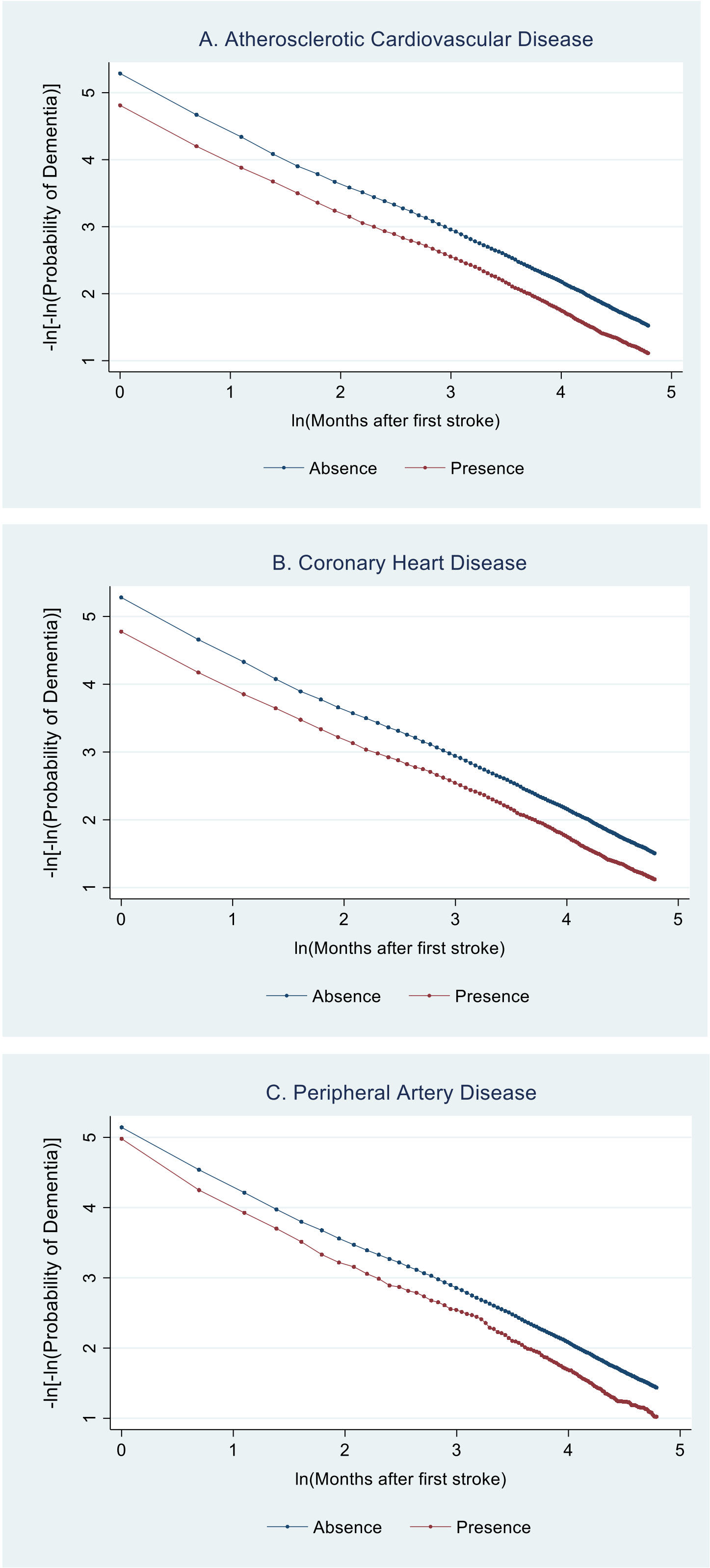

The RECORD statement – checklist of items, extended from the STROBE statement

	Item No.	STROBE items	Location in manuscript where items are reported	RECORD items	Location in manuscript where items are reported
**Title and abstract**					
	1	(a) Indicate the study's design with a commonly used term in the title or the abstract<br>(b) Provide in the abstract an informative and balanced summary of what was done and what was found	(a) Abstract<br><br>(b) Abstract	RECORD 1.1: The type of data used should be specified in the title or abstract. When possible, the name of the databases used should be included.<br><br>RECORD 1.2: If applicable, the geographic region and timeframe within which the study took place should be reported in the title or abstract.<br><br>RECORD 1.3: If linkage between databases was conducted for the study, this should be clearly stated in the title or abstract.	RECORD 1.1: Abstract<br><br>RECORD 1.2: Abstract<br><br>RECORD 1.3: Abstract
**Introduction**					
Background rationale	2	Explain the scientific background and rationale for the investigation being reported	The 1st to 4th paragraphs in the Introduction		
Objectives	3	State specific objectives, including any prespecified hypotheses	The 4th paragraph in the Introduction		
**Methods**					
Study Design	4	Present key elements of study design early in the paper	Key elements including "Study population", "Post-stroke dementia", "Exposure" and "Potential confounders"		
Setting	5	Describe the setting, locations, and relevant dates, including periods of recruitment,	All have been described in the Materials and Methods: setting		

		exposure, follow-up, and data collection	(general practices and hospitals), location (UK), dates (1 Jan 2006 to 31 Dec 2017), exposure (see "Exposure"), follow-up (10 years) and data collection (from the CPRD, HES, ONS, and IMD).		
Participants	6	*(a) Cohort study* - Give the eligibility criteria, and the sources and methods of selection of participants. Describe methods of follow-up<br>*Case-control study* - Give the eligibility criteria, and the sources and methods of case ascertainment and control selection. Give the rationale for the choice of cases and controls<br>*Cross-sectional study* - Give the eligibility criteria, and the sources and methods of selection of participants<br><br>*(b) Cohort study* - For matched studies, give matching criteria and number of exposed and unexposed<br>*Case-control study* - For matched studies, give matching criteria and the number of controls per case	*(a) Cohort study* – the first two paragraphs in the Materials and Methods	RECORD 6.1: The methods of study population selection (such as codes or algorithms used to identify subjects) should be listed in detail. If this is not possible, an explanation should be provided.<br><br>RECORD 6.2: Any validation studies of the codes or algorithms used to select the population should be referenced. If validation was conducted for this study and not published elsewhere, detailed methods and results should be provided.<br><br>RECORD 6.3: If the study involved linkage of databases, consider use of a flow diagram or other graphical display to demonstrate the data linkage process, including the number of individuals with linked data at each stage.	RECORD 6.1: Stroke codes were provided in Supplementary Table 1.<br><br>RECORD 6.2: The methods were specified on the website provided in the "Potential confounders" section in the Materials and Methods.<br><br>RECORD 6.3: Supplementary Figure 1.
Variables	7	Clearly define all	In the sections	RECORD 7.1: A	RECORD 7.1: The

		outcomes, exposures, predictors, potential confounders, and effect modifiers. Give diagnostic criteria, if applicable.	of "Post-stroke dementia", "Exposure" and "Potential confounders" in the Materials and Methods.	complete list of codes and algorithms used to classify exposures, outcomes, confounders, and effect modifiers should be provided. If these cannot be reported, an explanation should be provided.	codes for the outcome (post-stroke dementia) are listed in Supplementary Table 2. Codes for the exposures are listed in all the factors of interest were listed in Supplementary Tables 3 and 4. Codes for potential confounders were listed on the website provided in the "Potential confounders" section in the Materials and Methods.
Data sources/ measurement	8	For each variable of interest, give sources of data and details of methods of assessment (measurement). Describe comparability of assessment methods if there is more than one group	The 1st paragraph in the Materials and Methods.		
Bias	9	Describe any efforts to address potential sources of bias	Specified in each paragraph in the Methods, for example, rigorous procedure in codes development, quality control in data cleansing, examining proportional hazards assumption, and coping with missing data.		
Study size	10	Explain how the study size was arrived at	In the section of "Study		

			population" in the Materials and Methods. We included all eligible patients from the CPRD.		
Quantitative variables	11	Explain how quantitative variables were handled in the analyses. If applicable, describe which groupings were chosen, and why	In the section of "Potential confounders" in the Materials and Methods.		
Statistical methods	12	(a) Describe all statistical methods, including those used to control for confounding<br>(b) Describe any methods used to examine subgroups and interactions<br>(c) Explain how missing data were addressed<br>(d) *Cohort study* - If applicable, explain how loss to follow-up was addressed<br>*Case-control study* - If applicable, explain how matching of cases and controls was addressed<br>*Cross-sectional study* - If applicable, describe analytical methods taking account of sampling strategy<br>(e) Describe any sensitivity analyses	(a)-(e) The section of "Statistical analysis" in the Materials and Methods.		
Data access and cleaning methods		..		RECORD 12.1: Authors should describe the extent to which the investigators had access to the database population used to create the study population.<br><br>RECORD 12.2: Authors should provide information on the data cleaning methods used in the study.	RECORD 12.1: the section of "Data source" in the Methods.<br><br>RECORD 12.2: The section of "Statistical analysis" in the Materials and Methods and Supplementary

					Table 5.
Linkage		..		RECORD 12.3: State whether the study included person-level, institutional-level, or other data linkage across two or more databases. The methods of linkage and methods of linkage quality evaluation should be provided.	RECORD 12.3: Supplementary Figure 1.
**Results**					
Participants	13	(a) Report the numbers of individuals at each stage of the study (*e.g.*, numbers potentially eligible, examined for eligibility, confirmed eligible, included in the study, completing follow-up, and analysed)<br>(b) Give reasons for non-participation at each stage.<br>(c) Consider use of a flow diagram	(a) The first two paragraphs in the Results and Supplementary Figure 1.<br><br>(b) Supplementary Figure 1.<br>(c) Supplementary Figure 1.	RECORD 13.1: Describe in detail the selection of the persons included in the study (*i.e.,* study population selection) including filtering based on data quality, data availability and linkage. The selection of included persons can be described in the text and/or by means of the study flow diagram.	RECORD 13.1: Supplementary Figure 1.
Descriptive data	14	(a) Give characteristics of study participants (*e.g.*, demographic, clinical, social) and information on exposures and potential confounders<br>(b) Indicate the number of participants with missing data for each variable of interest<br>(c) *Cohort study* - summarise follow-up time (*e.g.*, average and total amount)	(a) Table 1 and Supplementary Tables 6 to 8 .<br><br>(b) The footnotes of Table 1.<br><br>(c) *Cohort study* – the first two paragraphs in the Results.		
Outcome data	15	*Cohort study* - Report numbers of outcome events or summary measures over time<br>*Case-control study* - Report numbers in each exposure category, or summary measures of exposure<br>*Cross-sectional study* -	*Cohort study* – the first two paragraphs in the Results, Table 2, and Figures 1 to 3.		

		Report numbers of outcome events or summary measures			
Main results	16	(a) Give unadjusted estimates and, if applicable, confounder-adjusted estimates and their precision (e.g., 95% confidence interval). Make clear which confounders were adjusted for and why they were included<br>(b) Report category boundaries when continuous variables were categorized<br>(c) If relevant, consider translating estimates of relative risk into absolute risk for a meaningful time period	(a) Table 2 and Figures 1 to 3.<br><br>(b) Figures 1 to 3.<br><br>(c) Not applicable.		
Other analyses	17	Report other analyses done—e.g., analyses of subgroups and interactions, and sensitivity analyses	Table 3 and Supplementary Tables 6 to 16 and Supplementary Figures 2 and 3.		
**Discussion**					
Key results	18	Summarise key results with reference to study objectives	The 1st paragraph in the Discussion		
Limitations	19	Discuss limitations of the study, taking into account sources of potential bias or imprecision. Discuss both direction and magnitude of any potential bias	The Strengths and limitations section.	RECORD 19.1: Discuss the implications of using data that were not created or collected to answer the specific research question(s). Include discussion of misclassification bias, unmeasured confounding, missing data, and changing eligibility over time, as they pertain to the study being reported.	RECORD 19.1: The Strengths and limitations section.
Interpretation	20	Give a cautious overall interpretation of results considering objectives, limitations, multiplicity of analyses, results from	The 4th to 8th paragraphs of the Discussion.		

		similar studies, and other relevant evidence			
Generalisability	21	Discuss the generalisability (external validity) of the study results	Specified in the strength of CPRD regarding its representativeness.		
**Other Information**					
Funding	22	Give the source of funding and the role of the funders for the present study and, if applicable, for the original study on which the present article is based	Specified in the Sources of Funding section.		
Accessibility of protocol, raw data, and programming code		..		RECORD 22.1: Authors should provide information on how to access any supplemental information such as the study protocol, raw data, or programming code.	When applicable, the links to any relevant information or any supplementary were provided in the Methods or Results.

*Reference: Benchimol EI, Smeeth L, Guttmann A, Harron K, Moher D, Petersen I, Sørensen HT, von Elm E, Langan SM, the RECORD Working Committee. The REporting of studies Conducted using Observational Routinely-collected health Data (RECORD) Statement. *PLoS Medicine* 2015; in press.

*Checklist is protected under Creative Commons Attribution (CC BY) license.
